# Health outcomes of smoking during pregnancy and the postpartum period: an umbrella review

**DOI:** 10.1186/s12884-021-03729-1

**Published:** 2021-03-26

**Authors:** Tuba Saygın Avşar, Hugh McLeod, Louise Jackson

**Affiliations:** 1 Health Economics Unit, University of Birmingham, Edgbaston, Birmingham, B15 2TT UK; 2 Population Health Sciences, Bristol Medical School, University of Bristol, Bristol, BS1 2NT UK; 3 National Institute for Health Research Applied Research Collaboration (NIHR ARC) West at University Hospitals Bristol and Weston NHS Foundation Trust, Bristol, UK

**Keywords:** Smoking during pregnancy, Partner smoking, Systematic review, Umbrella review, Health outcomes

## Abstract

**Background:**

Smoking during pregnancy (SDP) and the postpartum period has serious health outcomes for the mother and infant. Although some systematic reviews have shown the impact of maternal SDP on particular conditions, a systematic review examining the overall health outcomes has not been published. Hence, this paper aimed to conduct an umbrella review on this issue.

**Methods:**

A systematic review of systematic reviews (umbrella review) was conducted according to a protocol submitted to PROSPERO (CRD42018086350). CINAHL, EMBASE, MEDLINE, PsycINFO, Web of Science, CRD Database and HMIC databases were searched to include all studies published in English by 31 December 2017, except those focusing exclusively on low-income countries. Two researchers conducted the study selection and quality assessment independently.

**Results:**

The review included 64 studies analysing the relationship between maternal SDP and 46 health conditions. The highest increase in risks was found for sudden infant death syndrome, asthma, stillbirth, low birth weight and obesity amongst infants. The impact of SDP was associated with the number of cigarettes consumed. According to the causal link analysis, five mother-related and ten infant-related conditions had a causal link with SDP. In addition, some studies reported protective impacts of SDP on pre-eclampsia, hyperemesis gravidarum and skin defects on infants. The review identified important gaps in the literature regarding the dose-response association, exposure window, postnatal smoking.

**Conclusions:**

The review shows that maternal SDP is not only associated with short-term health conditions (e.g. preterm birth, oral clefts) but also some which can have life-long detrimental impacts (e.g. obesity, intellectual impairment).

**Implications:**

This umbrella review provides a comprehensive analysis of the overall health impacts of SDP. The study findings indicate that while estimating health and cost outcomes of SDP, long-term health impacts should be considered as well as short-term effects since studies not including the long-term outcomes would underestimate the magnitude of the issue. Also, interventions for pregnant women who smoke should consider the impact of reducing smoking due to health benefits on mothers and infants, and not solely cessation.

**Supplementary Information:**

The online version contains supplementary material available at 10.1186/s12884-021-03729-1.

## Background

Smoking during pregnancy (SDP) is a significant public health concern due to adverse health outcomes on mothers and infants, such as miscarriage, low birth weight (LBW), preterm birth, and asthma [1–4]. The prevalence of SDP is around 10% in high-income countries (HICs) [5–7] and 3% in low- and middle-income countries (LMICs) [8].

Smoking during pregnancy generates a considerable cost burden and the annual cost of smoking-related pregnancy complications has been estimated to be between £8 and £64 million in the UK, depending on the estimation method chosen [9]. In addition, the costs associated with the health problems experienced by the infant during the first year following the birth were found to be between £12 and £23 million [9]. Smoking during pregnancy poses a considerable economic burden in the USA as well, since smoking-attributable neo-natal costs were estimated to be nearly $228 million in total [10]. When long-term impacts on the infant are considered, the actual figures are likely to be higher. Therefore, to have a comprehensive estimate of the health and cost impacts of SDP to inform policy decisions and ensure that scarce health resources are allocated optimally, it is necessary to review the evidence on the overall health effects for mothers and infants over the longer term.

A scoping review and a review of reviews by Godfrey and colleagues [9], and a scoping review by Jones [11] provided a picture of the health and cost outcomes associated with SDP, and several narrative reviews about the health outcomes have been published [12–15]. However, none of these papers were fully systematic and comprehensive. Moreover, a considerable number of systematic reviews have been published more recently on the impact of maternal SDP on separate health outcomes, which makes this overall review of the current evidence timely.

The present study aimed to investigate the overall health impacts of maternal smoking during pregnancy and the postpartum period on mothers and infants. Additionally, the evidence on the impact of the number cigarettes consumed and second-hand smoking (SHS) by partner during pregnancy was assessed [16, 17].

## Methods

The guideline provided by the Cochrane Handbook for Systematic Reviews of Interventions [18] was followed. The review was carried out according to a protocol which included a detailed description of the methodology [19]. Umbrella reviews have been increasingly used to summarise the existing evidence on an issue by analysing all systematic reviews conducted [18, 20]. Considering the large number of original studies about health outcomes of SDP, an umbrella review was the appropriate design for this research.

Searches were undertaken of CINAHL, EMBASE, MEDLINE, PsycINFO, Web of Science, CRD Database (includes DARE, NHSEED and HTA) and HMIC databases. The search strategy for MEDLINE is presented in the Additional file 1. All systematic reviews published in English and by December 2017. Two independent reviewers conducted the study selection and quality assessment. The data extraction toll is provided in the Additional file 1: Table S1. The quality of included studies was assessed with a tool developed from the Centre for Reviews and Dissemination (CRD) checklist, which covers a range of issues including prior protocol use, bias in study selection, and consideration of publication bias and inclusion of a quality assessment [21]. Main outcome measures were odds ratios and relative risks for smoking women and their children compared to non-smoking women and their children.

To evaluate the causal link between SDP and the identified conditions which were found to have an association with SDP, a causal link analysis was conducted using established methods [11]. The evidence on the identified conditions was assessed and categorised using the following criteria:

- Strong evidence - one systematic review with ≥8 studies (group 1) or more than one systematic review (group 2);
- Weak evidence – more than one systematic review reported conflicting findings (group 3) or one systematic review reported limited number of studies (< 8) which found a relationship (group 4).

A validity assessment was conducted by reducing the threshold of eight studies to seven, and increasing it to 10 and 12. As discussed by Jones [11], this strength of evidence analysis fulfilled five of the nine items proposed by Hill [22] as conditions of a causal link (strength, consistency, specificity, temporality, and plausibility). In addition, the dose-response association was also considered. The remaining requirements (coherence, experiment, and analogy) of the Hill [22] criteria were irrelevant to this review as laboratory studies were not included and no causes other than smoking of the identified conditions were considered.

## Results

**Fig. 1:**
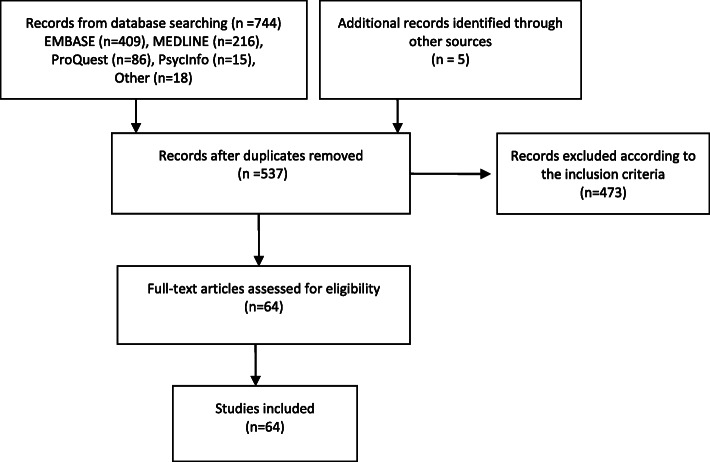
PRISMA Diagram for Study Selection

The database search yielded 744 studies and an additional five studies were found through hand searching the references of included studies. Following the removal of duplicates and abstract screening, 64 studies were selected for full-text analysis Fig. 1.

### Characteristics of the included studies

Most reviews (*n* = 46) were published since 2010. Only 13 reviews investigated a health condition related to mothers; the other 49 reviews analysed infant-related conditions, except two [23, 24] which evaluated the impacts on both. Key characteristics of the included reviews are provided in Additional file 1: Table S2.

In most reviews (*n* = 27 reviews), the included studies were predominantly from HICs, and 22 of the included reviews covered studies from HIC only. In two reviews [3, 25] most of the included studies were concerned with upper-middle-income countries.

In 12 reviews, the country of focus of the included studies was not provided. However, one of them [26] conducted a meta-analysis of the studies from Europe only, and in five reviews, the language of the included studies was either only English [27–29] or languages [30, 31] which are only spoken by HICs. In the remaining six reviews [32–36] there was no indication of whether the studies focussed on LMICs or HICs. Nevertheless, when interpreting the results of these reviews, the possibility that studies which were conducted in LMICs have been included in addition to HICs should be born in mind.

### Quality of the included studies

The quality scores of the reviews are provided in Additional file 1: Table S2. The highest achievable score was 16, and most reviews (*n* = 46) scored between nine and 14 while two reviews [25, 32] achieved very low scores of 4 and 5. Therefore, most of the included reviews were moderate or high quality studies according to the criteria used.

Study selection was made by two reviewers independently in almost half of the reviews (*n* = 31) to minimise bias. The majority of the studies (*n* = 50) assessed publication bias. Heterogeneity was measured in all reviews although causes of heterogeneity were not analysed in some (*n* = 17). However, only seven reviews reported protocol publication [3, 26, 33, 37–40].

### Impacts of smoking during pregnancy on mothers

Overall, of the 14 reviews that reported the impact of smoking on mothers, all except two [41, 42] conducted meta-analyses (Additional file 1: Table S3). The reviews presented consistent findings, suggesting a significantly increased risk associated with smoking and seven health conditions. The highest risks were reported for spontaneous miscarriage in assisted reproduction (OR = 2.65, 95% CI, 1.33–5.30, 28) and ectopic pregnancy (OR = 2.30, 95% CI, 2.02–2.80, 30). Two conditions (preeclampsia and hypremesis gravidarum) were found to be negatively associated with SDP. Hence, women who smoked whilst pregnant were less likely to experience these two conditions.

### Impacts of smoking during pregnancy on infants

Studies found a smoking-related increased risk for 20 conditions and the highest impact was observed for sudden infant death syndrome (SIDS) (OR = 2.98, 95% CI, 2.51–3.54) [24], asthma (OR = 1.85, 95% CI, 1.35–2.53) [1], LBW (OR = 1.75, 95% CI, 1.42–2.10), stillbirth (OR = 1.55, 95% CI, 1.36–1.78) [38] and obesity (OR = 1.60, 95% CI 1.37–1.88) [43]. Studies did not find any significant association between 15 conditions and SDP, including autism, brain tumors, breast cancer in daughters and testicular cancer in sons. On the other hand, a protective impact on skin defects was observed in one review [44].

Most studies (*n* = 42) investigating the impacts of SDP on infants conducted a meta-analysis (Additional file 1: Table S4), and only nine did not include this (Additional file 1: Table S5). In these studies, there was no significant relationship between maternal SDP and lung functions, or Tourette’s syndrome.

The age group of study participants varied between studies; for example, some conditions were assessed amongst infants while some were measured in adults. In some reviews, participants were both infants and adults. Table 1 lists health conditions by the life stage they were assessed.

**Table 1:** Health impacts of maternal SDP on infants by life stages

Life Stage	Health outcomes
**Perinatal**	Foetal loss in assisted reproduction, stillbirth, perinatal death, preterm birth [4, 38, 45, 46]
**Neonatal**	Accelerated growth, birth defects (i.e. heart defects, oral clefts), LBW, neonatal death, neural tube defects, sleep apnoea [3, 23, 24, 39, 44, 46–51]
**Infancy**	Asthma, cognitive parameters impairment (decreased IQ, impaired neurodevelopment), intellectual disability, lower respiratory illness (LRI), SIDS, wheezing [1, 37, 42, 52–54]
**Childhood**	ADHD, accelerated growth, asthma, central nervous system tumours, cognitive parameters impairment (decreased IQ), disruptive behaviour disorders, intellectual disability, neuroblastoma, non-Hodgkin lymphoma, overweight, obesity, vision difficulties, wheezing [1, 23, 27, 43, 52–60]
**Adolescence**	Asthma, Central nervous system tumours, cognitive impairment (decreased IQ, poor school performance), neuroblastoma, overweight, obesity, reduced menarche age in daughters, wheezing [1, 43, 53, 54, 57, 61]
**Adulthood**	Central nervous system tumours, cognitive parameters impairment (decreased IQ), overweight, obesity [44, 54, 56, 58]

The reviews included in this study indicated that maternal smoking increased the risk of death for the child during the prenatal period, neonatal period and infancy. The evidence showed maternal SDP did not only have short-term impact but also some long-term outcomes which could be detrimental for offspring. Moreover, some of the conditions measured in early life stages could continue later in life. For instance, some birth defects and intellectual disability would affect later stages of life.

### Dose-response association

To understand the impact of reductions in smoking, the relationship between the number of cigarettes consumed and the health implications for infants or mothers were investigated. Although a dose-response impact of SDP was reported in 27 reviews (22 related to infant conditions), it was statistically tested in just 17 studies. Among them, four found no significant impact of SDP and their dose-response tests showed similar results. In addition, one review [62] reported a dose-response association for SIDS but did not provide the odds ratios. Findings of the remaining 12 studies are summarised in the Additional file 1: Table S6.

To define light, moderate and heavy smokers, most studies [38, 39, 46, 62–64] chose smoking 10 cigarettes daily as a cut-off point to distinguish light smokers from moderate and heavy smokers. In some studies [4, 39, 46, 61, 64], both 10 cigarettes daily and 20 cigarettes daily were utilised as the thresholds. In one review the number of cigarettes consumed daily for each category was inconsistent [65]. All studies estimated the risk ratios compared to non-smokers [66], except for one review, in which light smokers were compared to moderate smokers.

Included reviews showed that the risk of stillbirth, birth defects, preterm birth and perinatal death elevated as the number of cigarettes increased [4, 38, 39, 46]. In contrast, smoking not only protected against pre-eclampsia but the risk reduced as exposure increased [67].

A dose-response relationship was found in five reviews although a pooled estimation was not calculated. They reported an increased risk for placental abruption [68], and for the offspring the risk of being overweight [57], having oral clefts [29, 50], or a decrease in cognitive abilities [53] increased along with the number of cigarettes that the mothers consumed. Five reviews included studies reporting a dose-response relationship along with others that did not find any relationship [1, 41, 51, 56, 69]. Therefore, it was not clear whether or not the risk for some conditions (pre-eclampsia, and in the offspring asthma, attention deficit hyperactivity disorder, and vision difficulties) was affected by the number of cigarettes consumed.

Six reviews observed no significant association between the number of cigarettes consumed and the risk of health conditions for the children exposed to maternal SDP, although overall they reported a significantly increased risk. These studies covered congenital heart diseases [65], central nervous system tumors [64], childhood neuroblastoma [63], lower respiratory infections (LRI) [37] and lymphoblastic leukaemia [66], and reduced menarche age in daughters [61].

### Impacts of postnatal maternal smoking on infants

The main findings of the reviews which investigated the impact of postnatal smoking on the infants are shown in Additional file 1: Table S7. The reviews showed an increased impact on asthma, LRI, SIDS and wheezing but not on leukaemia and obesity. However, in some studies, it was not clear whether or not the mothers included in the studies smoked during the whole pregnancy as well as the postpartum period. This is a significant consideration as one study reported by Oken et al. [57] found no increase in the prevalence of obesity when the mother smoked only after birth, whereas smoking before and throughout pregnancy were found to be related with an increased risk [70].

### Impact of second-hand smoking by partners

In addition to active smoking, SHS during pregnancy could have health implications. It was important to understand whether the health-related risks were higher when partners smoked during pregnancy. Therefore, partner-related findings of the included reviews were analysed. Partner smoking was considered in only 12 reviews of which six did not assess the impact of SHS specific to the pregnancy period (Additional file 1: Table S8). None of the studies reported the combined impact of SDP and SHS by the partner during pregnancy. Two reviews reported an increased risk of SIDS [71] and delay in mental development [25] when the partners of non-smoking women smoked during pregnancy, while no association was found for brain tumors [72] or breast cancer risk in daughters [73].

### Sub-group analyses in the included reviews

The reviews conducted sub-group analyses to assess the impact of study design, sample size, the duration of the infant exposure to smoking (i.e. pre-pregnancy, first trimester or the whole pregnancy) and adjustments for confounding factors. The study findings did not differ significantly in most of the analyses except for adjustments for confounding and study quality. The evidence was not sufficient to make a comparison based on country income groups because most studies were from high-income countries.

Although the included meta-analyses utilised the most adjusted estimations of observational studies when pooling their results, only 10 of the included reviews provided risk ratios for adjusted and unadjusted estimations (Additional file 1: Table S9). Studies with unadjusted ratios estimated greater values for miscarriage, perinatal death, SDIS, overweight and obesity.

Sub-group analyses based on quality appraisal of the included studies were conducted in 14 reviews (Additional file 1: Table S10). The results showed that high-quality studies reported higher ratios for some conditions (overweight, obesity, placenta previa) as opposed to lower or insignificant ratios for some others (e.g. LBW, miscarriage, stillbirth).

Two reviews [46, 74] compared the type of smoking status data and found similar results for biochemical and self-reported data. The exposure period was researched in five reviews [40, 41, 46, 64, 75], and the results showed no significant difference between women who quit early in pregnancy and those who did not smoke.

### Causal link analysis

The causal link analysis identified a range of health conditions found to have strong association with SDP; these are presented in Table 2, grouped according to the strength of evidence.

**Table 2:** Strength of evidence analysis

Groups	Health conditions
Strong evidence (group 1)	Miscarriage, hyperemesis gravidarum, LRI, neonatal death, and preterm birth, reduced live birth in assisted reproduction
Strong evidence (group 2)	Abruptio placenta, ectopic pregnancy, placenta previa, pre-eclampsia,birth defects (except skin defect), asthma, LBW, overweight, obesity, SIDS, stillbirth.
Weak evidence (group 3)	Leukaemia, lymphoma, vision difficulties
Weak evidence (group 4)	Cognitive parameters of children and young adults, disruptive behaviour disorders, intellectual disability, neuroblastoma, preterm premature rupture of the membranes (PPROM), reduced menarche age, sleep apnea, skin defects, Tourette’s syndrome.

Nearly all of the conditions for which a strong association was identified fulfilled the criteria for a causal link. The health conditions were largely reported by moderate- or high-quality reviews and there were consistent findings in the sub-group analyses. There was not a sufficient biological explanation to the correlation found between hyperemesis gravidarum and SDP, hence although there was a strong association, a causal link could not be confirmed.

## Discussion

This study analysed the health impacts of smoking during pregnancy and during the postpartum period on mothers and infants. The 64 included reviews covered 1744 studies relating to SDP or smoking during the postpartum period. The review found that maternal SDP has short-term and long-term health consequences, suggesting a positive association between 20 infant-related and seven mother-related conditions, and a negative association with two maternal conditions. The review did not find a statistically significant impact of SDP on 15 infant-related conditions while conflicting findings were reported for leukaemia and lymphoma.

The causal link analysis of the conditions that were found to have an association with SDP suggested that five mother-related and 10 infant-related conditions had a causal link with SDP. PPROM and intellectual disability in children did not fulfil the criteria for the casual link although meta-analyses reported a statistically significant relationship with SDP.

### Health conditions with conflicting results

Some health conditions were assessed in multiple meta-analyses and they reported conflicting results. For instance, the increased risk of having any type of birth defect was statistically significant despite being small in the effect size (OR = 1.18, 95% CI, 1.14–1.22) in one review [39] as opposed to a borderline ratio (OR = 1.01, 95% CI, 0.96–1.07) reported in another [44]. The main difference was the reduced risk of skin defects (OR = 0.82, 95% CI, 0.75–0.89) which was included in the latter [44] while omitted in the former [39] without any justification. All five studies included in this meta-analysis reported a negative relationship and the heterogeneity was low (*P* = 0.00001, I^2^ = 0%). Therefore, the evidence suggested an increased risk of birth defects except for skin defects amongst SDP exposed children. However, there was no biological explanation for the potential protective impact of SDP on skin defects.

Another health condition with mixed findings was leukaemia. One meta-analysis [64] including 19 studies indicated an insignificant decreased risk (OR = 0.99, 95% CI, 0.92–1.06) whereas another review [66] of 21 studies found an increased risk (OR = 1.10, 95% CI, 1.02–1.19). The difference could be explained by the different studies included, since there were only five studies common to both, and the association between SDP and leukaemia is unclear.

Similarly, the reviews reported different results for lymphoma. One meta-analysis [55] found an insignificant association between any lymphoma and SDP based on eight studies (OR = 1.10, 95% CI, 0.96–1.27), although positive relationship for non-Hodgkin lymphoma was reported (OR = 1.22, 95% CI, 1.03–1.45, *n* = 8). Another review [64] which included six studies found an increased risk for any lymphoma (OR = 1.21, 95% CI, 1.05–1.34). Hence, SDP increases the risk of non-Hodgkin lymphoma but for other types of lymphoma the impact is unclear.

### Strengths and limitations of the umbrella review

To the best of the authors’ knowledge, this is the first umbrella review on the topic and provides the most systematic and comprehensive assessment of the current evidence. The criteria to assess any causal links are an important consideration. The tool developed by Hill [22] is widely recognised for assessing causation. In addition to these criteria, this study considered the quality of reviews and the findings of sub-group analyses. Hence, the conditions identified by the causal link analysis are very likely to have a causal link with SDP.

The review has some limitations. Firstly, although systematic reviews are accepted as the highest in the evidence hierarchy [76, 77], the focus on systematic reviews alone meant some health conditions were not covered. Some original studies have indicated the impact of SDP on other infant-related conditions, such as diabetes [78], hypomania [79], otitis [80] and pervasive development disorder [81], which were not assessed in a systematic review, and as a result were not included in this study. Furthermore, SDP has been shown to be related to the smoking uptake of the offspring [82, 83]. There are also some maternal health conditions found to be related to smoking whilst pregnant in one study; vein thrombosis, myocardial infarction, influenza or pneumonia, bronchitis, gastrointestinal ulcers [84]. However, the current study focused on the conditions for which there was strong evidence from systematic reviews.

The methodological limitations of the original studies covered in the included reviews should be born in mind when interpreting the results of the current review. First, long-term implications of SDP were often tested retrospectively by asking mothers whether or not they had smoked during pregnancy. This clearly has limitations as these studies were not designed to compare the offspring of smoking mothers with the children of non-smoking mothers to determine differences in their health, but rather to compare the exposure in children with particular conditions and those without these conditions. The second issue is the usual reliance on mother’s memory and openness about their smoking behaviour is unsatisfactory. The third issue is the impact of confounding factors. For example, a seven-year-old child with diagnosed asthma could have a mother who smoked during pregnancy only and a father who smoked during pregnancy and the postpartum period. To minimise the impact of this the most adjusted estimations were reported in this review.

### The review in the context of literature

Two previous scoping reviews were conducted to define the health outcomes of SDP although they did not focus on systematic reviews [9, 11]. The scoping review by Jones et al. [11] was more comprehensive and included 32 health conditions. A quality assessment was not conducted but specific criteria were used to assess the strength of the evidence. According to the criteria, Jones et al. suggested that the evidence for a link between obesity and SDP was not strong [11]. However, the current analysis suggests a causal link due to the inclusion of two subsequently published systematic reviews [32, 43].

Some of the health conditions covered in this study were also included in the review by Godfrey et al. but often higher ratios were reported [9]. This might be because they included narrative reviews which did not separate maternal SDP and postnatal passive smoke exposure while estimating the summary risk ratios [24, 85–87]. Moreover, none of the previous reviews analysed the impact of the number of cigarettes consumed, partners’ smoking and postpartum smoking on infants. Therefore, the current review is more comprehensive and more systematic than previous studies.

### Gaps in the literature

The study identified important gaps in the literature which warrant further research. In particular, there is a need to further our understanding of dose-response association, the impact of postnatal smoking, and SHS during pregnancy. Current evidence on the impact of number of cigarettes consumed suggests that even low amounts of cigarette consumption during pregnancy have significant health outcomes and there is a clear gradient for some conditions. This indicates the importance of smoking cessation during pregnancy and if reduction in smoking which is often not addressed in smoking cessation interventions designed for pregnant women.

Only two studies assessed the impact of SHS by partners during pregnancy when the mother was a non-smoker. There was no review reporting the combined impact of SDP and SHS by partners during pregnancy while two reviews reported increased risks for SID [43] and delay in mental development [25] when only the partner smoked during pregnancy. Hence, more research is needed to understand the impacts of having a smoking partner during pregnancy.

## Conclusion

This study has shown that smoking during pregnancy and the postpartum period has significant health consequences for mothers and infants. It is important to encourage pregnant smokers to quit smoking or reduce the number of cigarettes consumed if they are not prepared to quit entirely since the existing evidence indicates a dose-response association. Similarly, the impact of SHS needs to be considered to promote a smoke-free environment for the mother and infant.

## Supplementary Information

**Additional file 1.** MEDLINE Search strategy and Tables S1-S10.

## Data Availability

Not applicable.

## Abbreviations

SDP: Smoking during pregnancy
LBW: Low birth weight
LRI: Lower respiratory infections
HICs: High-income countries
LMICs: Low and middle-income countries
CRD: Centre for Reviews and Dissemination
SHS: Second-hand smoking
SIDS: Sudden infant death syndrome

## References

[1] Burke H, Leonardi-Bee J, Hashim A, Pine-Abata H, Chen Y, Cook DG, et al. Prenatal and passive smoke exposure and incidence of asthma and wheeze: systematic review and Meta-analysis. Pediatrics. 2012;129(4):735–44. 10.1542/peds.2011-2196. 10.1542/peds.2011-2196 22430451

[2] Castles A, Adams EK, Melvin CL, Kelsch C, Boulton ML (1999). Effects of smoking during pregnancy. Five meta-analyses. Am J Prev Med.

[3] Pereira PP, Da Mata FA, Figueiredo AC, de Andrade KR, Pereira MG (2017). Maternal active smoking during pregnancy and low birth weight in the Americas: a systematic review and Meta-analysis. Nicotine Tob Res.

[4] Shah NR, Bracken MB (2000). A systematic review and meta-analysis of prospective studies on the association between maternal cigarette smoking and preterm delivery. Am J Obstet Gynecol.

[5] Australian Institute of Health and Welfare (2017). Australia’s mothers and babies 2015.

[6] NHS (2017). Statistics on Smoking.

[7] Tong VT, Dietz PM, Farr SL, D'Angelo DV, England LJ (2013). Estimates of smoking before and during pregnancy, and smoking cessation during pregnancy: comparing two population-based data sources. Public Health Rep (Washington, DC : 1974).

[8] Caleyachetty R, Tait CA, Kengne AP, Corvalan C, Uauy R, Echouffo-Tcheugui JB (2014). Tobacco use in pregnant women: analysis of data from demographic and health surveys from 54 low-income and middle-income countries. Lancet Glob Health.

[9] Godfrey C, Pickett KE, Parrott S, Mdege ND, Eapen D (2010). Estimating the costs to the NHS of smoking in pregnancy for pregnant women and infants.

[10] CDC (2002). Annual Smoking-Attributable Mortality, Years of Potential Life Lost, and Economic Costs, United States.

[11] Jones M (2015). The development of the economic impacts of smoking in pregnancy (ESIP) model for measuring the impacts of smoking and smoking cessation during pregnancy: University of Nottingham.

[12] Banderali G, Martelli A, Landi M, Moretti F, Betti F, Radaelli G, et al. Short and long term health effects of parental tobacco smoking during pregnancy and lactation: a descriptive review. J Transl Med. 2015;13(1):327. 10.1186/s12967-015-0690-y. 10.1186/s12967-015-0690-y PMC4608184 26472248

[13] Bruin JE, Gerstein HC, Holloway AC (2010). Long-term consequences of fetal and neonatal nicotine exposure: a critical review. Toxicol Sci.

[14] Delpisheh A, Brabin L, Brabin BJ (2006). Pregnancy, smoking and birth outcomes. Women Health.

[15] Cnattingius S (2004). The epidemiology of smoking during pregnancy: Smoking prevalence, maternal characteristics, and pregnancy outcomes. Nicotine Tob Res.

[16] NICE (2010). Smoking: stopping in pregnancy and after childbirth (PH26).

[17] Baxter S, Blank L, Guillaume L, Messina J, Everson-Hock E, Burrows J. Systematic review of how to stop smoking in pregnancy and following childbirth: University of Sheffield; 2009.

[18] Higgins J, Green S (2011). Cochrane handbook for systematic reviews of interventions version 5.1.0.

[19] Saygın Avşar T, McLeod H, Jackson L (2018). Health outcomes of maternal smoking during pregnancy and postpartum period for the mother and infant: protocol for an umbrella review. Syst Rev.

[20] Aromataris E, Fernandez R, Godfrey CM, Holly C, Khalil H, Tungpunkom P (2015). Summarizing systematic reviews: methodological development, conduct and reporting of an umbrella review approach. Int J Evid-Based Health.

[21] CRD (2009). CRD’s guidance for undertaking reviews in health care: Centre for Reviews and Dissemination, University of York.

[22] Hill AB (1965). The environment and disease: association or causation?. Proc R Soc Med.

[23] Jayes L, Haslam PL, Gratziou CG, Powell P, Britton J, Vardavas C, et al. SmokeHaz: systematic reviews and Meta-analyses of the effects of smoking on respiratory health. Chest. 2016;150(1):164–79. 10.1016/j.chest.2016.03.060. 10.1016/j.chest.2016.03.060 27102185

[24] Difranza JR, Lew RA (1995). Effect of maternal cigarette-smoking on pregnancy complications and sudden-infant-death-syndrome. J Fam Pract.

[25] Tsai MS, Chen MH, Lin CC, Ng S, Hsieh CJ, Liu CY, et al. Children’s environmental health based on birth cohort studies of Asia. Sci Total Environ. 2017;609:396–409. 10.1016/j.scitotenv.2017.07.081. 10.1016/j.scitotenv.2017.07.081 28755589

[26] Kantor R, Kim A, Thyssen JP, Silverberg JI (2016). Association of atopic dermatitis with smoking: A systematic review and meta-analysis. J Am Acad Dermatol.

[27] Latimer K, Wilson P, Kemp J, Thompson L, Sim F, Gillberg C (2012). Disruptive behaviour disorders: a systematic review of environmental antenatal and early years risk factors. Child Care Health Dev.

[28] Waylen AL, Metwally M, Jones GL, Wilkinson AJ, Ledger WL (2009). Effects of cigarette smoking upon clinical outcomes of assisted reproduction: a meta-analysis. Hum Reprod Update.

[29] Xuan Z, Zhongpeng Y, Yanjun G, Jiaqi D, Yuchi Z, Bing S, et al. Maternal active smoking and risk of oral clefts: a meta-analysis. Oral Surg Oral Med Oral Pathol Oral Radiol. 2016;122(6):680–90. 10.1016/j.oooo.2016.08.007. 10.1016/j.oooo.2016.08.007 27727103

[30] Ankum WM, Mol BWJ, Van der Veen F, Bossuyt PMM (1996). Risk factors for ectopic pregnancy: a meta-analysis. Fertil Steril.

[31] Chao TK, Hu J, Pringsheim T (2014). Prenatal risk factors for tourette syndrome: a systematic review. BMC Pregnancy Childbirth.

[32] Huang JS, Lee TA, Lu MC (2007). Prenatal programming of childhood overweight and obesity. Matern Child Health J.

[33] Huncharek MS, Kupelnick B, Klassen H (2002). Maternal smoking during pregnancy and the risk of childhood brain tumors: a meta-analysis of 6566 subjects from twelve epidemiological studies. J Neuro-Oncol.

[34] Ferrante G, Antona R, Malizia V, Montalbano L, Corsello G, La Grutta S (2014). Smoke exposure as a risk factor for asthma in childhood: a review of current evidence. Allergy Asthma Proceed.

[35] Little J, Cardy A, Munger RG (2004). Tobacco smoking and oral clefts: a meta-analysis. Bull World Health Organ.

[36] Tuomisto J, Holl K, Rantakokko P, Koskela P, Hallmans G, Wadell G, et al. Maternal smoking during pregnancy and testicular cancer in the sons: a nested case-control study and a meta-analysis. Eur J Cancer. 2009;45(9):1640–8. 10.1016/j.ejca.2009.01.017. 10.1016/j.ejca.2009.01.017 19231156

[37] Jones LL, Hashim A, McKeever T, Cook DG, Britton J, Leonardi-Bee J (2011). Parental and household smoking and the increased risk of bronchitis, bronchiolitis and other lower respiratory infections in infancy: systematic review and meta-analysis. Respir Res.

[38] Marufu TC, Ahankari A, Coleman T, Lewis S (2015). Maternal smoking and the risk of still birth: systematic review and meta-analysis. BMC Public Health.

[39] Nicoletti D, Appel LD, Siedersberger Neto P, Guimaraes GW, Zhang L. Maternal smoking during pregnancy and birth defects in children: a systematic review with meta-analysis. Cadernos de saude publica. 2014;30(12):2491–529. 10.1590/0102-311x00115813. 10.1590/0102-311X00115813 26247979

[40] Wendland EM, Pinto ME, Duncan BB, Belizán JM, Schmidt MI (2008). Cigarette smoking and risk of gestational diabetes: a systematic review of observational studies. BMC Pregnancy Childbirth.

[41] England L, Zhang J (2007). Smoking and risk of preeclampsia: a systematic review. Front Biosci.

[42] Lancaster CA, Gold KJ, Flynn HA, Yoo H, Marcus SM, Davis MM (2010). Risk factors for depressive symptoms during pregnancy: a systematic review. Am J Obstet Gynecol.

[43] Riedel C, Schonberger K, Yang S, Koshy G, Chen YC, Gopinath B, et al. Parental smoking and childhood obesity: higher effect estimates for maternal smoking in pregnancy compared with paternal smoking--a meta-analysis. Int J Epidemiol. 2014;43(5):1593–606. 10.1093/ije/dyu150. 10.1093/ije/dyu150 25080528

[44] Hackshaw A, Charles R, Sadie B (2011). Maternal smoking in pregnancy and birth defects: a systematic review based on 173 687 malformed cases and 11.7 million controls. Hum Reprod Update.

[45] Flenady V, Koopmans L, Middleton P, Froen J, Smith GC, Gibbons K (2011). Major risk factors for stillbirth in high-income countries: a systematic review and meta-analysis. Lancet.

[46] Pineles BL, Hsu S, Park E, Samet JM (2016). Systematic review and Meta-analyses of perinatal death and maternal exposure to tobacco smoke during pregnancy. Am J Epidemiol.

[47] Chrestani MA, Santos IS, Horta BL, Dumith SC, de Oliveira Dode MA (2013). Associated factors for accelerated growth in childhood: a systematic review. Matern Child Health J.

[48] Lee L, Lupo P (2012). Maternal smoking during pregnancy and risk of congenital heart defects: a systematic review and a meta-analysis. Circ Conf.

[49] Wang M, Wang ZP, Zhang M, Zhao ZT (2014). Maternal passive smoking during pregnancy and neural tube defects in offspring: a meta-analysis. Arch Gynecol Obstet.

[50] Wyszynski DF, Duffy DL, Beaty TH (1997). Maternal cigarette smoking and oral clefts: a meta-analysis. Cleft Palate Craniofac J.

[51] Zhang D, Cui H, Zhang L, Huang Y, Zhu J, Li X (2017). Is maternal smoking during pregnancy associated with an increased risk of congenital heart defects among offspring? A systematic review and meta-analysis of observational studies. J Matern-Fetal Neonatal Med.

[52] Brion M-JA, Leary SD, Lawlor DA, Smith GD, Ness AR (2008). Modifiable maternal exposures and offspring blood pressure: a review of epidemiological studies of maternal age, diet, and smoking. Pediatr Res.

[53] Clifford A, Lang L, Chen R (2012). Effects of maternal cigarette smoking during pregnancy on cognitive parameters of children and young adults: a literature review. Neurotoxicol Teratol.

[54] Ino T (2010). Maternal smoking during pregnancy and offspring obesity: Meta-analysis. Pediatr Int.

[55] Antonopoulos CN, Sergentanis TN, Papadopoulou C, Andrie E, Dessypris N, Panagopoulou P, et al. Maternal smoking during pregnancy and childhood lymphoma: a meta-analysis. Int J Cancer. 2011;129(11):2694–703. 10.1002/ijc.25929. 10.1002/ijc.25929 21225624

[56] Linnet KM, Dalsgaard S, Obel C, Wisborg K, Henriksen TB, Rodriquez A (2003). Maternal lifestyle factors in pregnancy risk of attention deficit hyperactivity disorder and associated behaviors: review of the current evidence. Am J Psychiatry.

[57] Oken E, Levitan E, Gillman M (2008). Maternal smoking during pregnancy and child overweight: systematic review and meta-analysis. Int J Obes.

[58] Rayfield S, Plugge E (2017). Systematic review and meta-analysis of the association between maternal smoking in pregnancy and childhood overweight and obesity. J Epidemiol Commun Health.

[59] Weng SF, Redsell SA, Swift JA, Yang M, Glazebrook CP (2012). Systematic review and meta-analyses of risk factors for childhood overweight identifiable during infancy. Arch Dis Child.

[60] Huang J, Zhu T, Qu Y, Mu D (2016). Prenatal, perinatal and neonatal risk factors for intellectual disability: a systemic review and meta- Analysis. PLoS One.

[61] Yermachenko A, Dvornyk V (2015). A meta-analysis provides evidence that prenatal smoking exposure decreases age at menarche. Reprod Toxicol.

[62] Zhang K, Wang X (2013). Maternal smoking and increased risk of sudden infant death syndrome: a meta-analysis. Legal Med (Tokyo, Japan).

[63] Chu P, Wang H, Han S, Jin Y, Lu J, Han W, et al. Maternal smoking during pregnancy and risk of childhood neuroblastoma: systematic review and meta-analysis. J Cancer Res Ther. 2016;12(2):999. 10.4103/0973-1482.171367 27461688

[64] Rumrich IK, Viluksela M, Vahakangas K, Gissler M, Surcel HM, Hanninen O (2016). Maternal smoking and the risk of cancer in early life - a meta-analysis. PLoS ONE [Electronic Resource].

[65] Lee LJ, Lupo PJ (2013). Maternal smoking during pregnancy and the risk of congenital heart defects in offspring: a systematic review and metaanalysis. Pediatr Cardiol.

[66] Yan K, Xu X, Liu X, Wang X, Hua S, Wang C, et al. The associations between maternal factors during pregnancy and the risk of childhood acute lymphoblastic leukemia: a meta-analysis.[erratum appears in Pediatr blood Cancer. 2016 may;63(5):953-4; PMID: 26999072]. Pediatr Blood Cancer. 2015;62(7):1162–70. 10.1002/pbc.25443. 10.1002/pbc.25443 25728190

[67] Conde-Agudelo A, Althabe F, Belizan JM, Kafury-Goeta AC (1999). Cigarette smoking during pregnancy and risk of preeclampsia: a systematic review. Am J Obstet Gynecol.

[68] Ananth CV, Smulian JC, Vintzileos AM (1999). Incidence of placental abruption in relation to cigarette smoking and hypertensive disorders during pregnancy: a meta-analysis of observational studies. Obstet Gynecol.

[69] Fernandes M, Yang X, Li JY, Cheikh IL (2015). Smoking during pregnancy and vision difficulties in children: a systematic review. Acta Ophthalmol.

[70] Toschke AM, Beyerlein A, von Kries R (2005). Children at high risk for overweight: a classification and regression trees analysis approach. Obes Res.

[71] Mitchell EA, Milerad J (2006). Smoking and the sudden infant death syndrome. Rev Environ Health.

[72] Huang Y, Huang J, Lan H, Zhao G, Huang C (2014). A meta-analysis of parental smoking and the risk of childhood brain tumors. PLoS One.

[73] Park SK, Kang D, McGlynn KA, Garcia-Closas M, Kim Y, Yoo KY (2008). Intrauterine environments and breast cancer risk: meta-analysis and systematic review. Breast Cancer Res.

[74] Jones LL, Hassanien A, Cook DG, Britton J, Leonardi-Bee J (2012). Parental smoking and the risk of middle ear disease in children: a systematic review and meta-analysis. Arch Pediatr Adolesc Med.

[75] Pineles BL, Park E, Samet JM (2014). Systematic review and Meta-analysis of miscarriage and maternal exposure to tobacco smoke during pregnancy. Am J Epidemiol.

[76] Evans D (2003). Hierarchy of evidence: a framework for ranking evidence evaluating healthcare interventions. J Clin Nurs.

[77] Murad MH, Asi N, Alsawas M, Alahdab F (2016). New evidence pyramid. Evid Based Med.

[78] Montgomery SM, Ekbom A (2002). Smoking during pregnancy and diabetes mellitus in a British longitudinal birth cohort. Br Med J.

[79] Mackay DF, Anderson JJ, Pell JP, Zammit S, Smith DJ (2017). Exposure to tobacco smoke in utero or during early childhood and risk of hypomania: prospective birth cohort study. Eur Psychiatry.

[80] Haberg SE, Bentdal YE, London SJ, Stigum H, Kvaerner KJ, Nystad W (2009). Pre- and postnatal parental smoking and acute otitis Media in Early Childhood. Am J Resp Crit Care.

[81] Tran PL, Lehti V, Lampi KM, Helenius H, Suominen A, Gissler M, et al. Smoking during pregnancy and risk of autism spectrum disorder in a Finnish National Birth Cohort. Paediatr Perinat Epidemiol. 2013;27(3):266–74. 10.1111/ppe.12043. 10.1111/ppe.12043 PMC3652271 23574415

[82] Biederman J, Martelon M, Woodworth KY, Spencer TJ, Faraone SV (2017). Is maternal smoking during pregnancy a risk factor for cigarette smoking in offspring? A longitudinal controlled study of ADHD children grown up. J Atten Disord.

[83] Niemela S, Raisanen A, Koskela J, Taanila A, Miettunen J, Ramsay H (2016). The effect of prenatal smoking exposure on daily smoking among teenage offspring. Addiction..

[84] Roelands J, Jamison MG, Lyerly AD, James AH (2009). Consequences of smoking during pregnancy on maternal health. J Women's Health.

[85] Andres RL, Day M-C (2000). Perinatal complications associated with maternal tobacco use. Semin Neonatol.

[86] Salihu HM, Wilson RE (2007). Epidemiology of prenatal smoking and perinatal outcomes. Early Hum Dev.

[87] Walsh RA, Redman S, Brinsmead MW, Byrne JM, Melmeth A (1997). A smoking cessation program at a public antenatal clinic. Am J Public Health.

