# Supplementary material for: Health outcomes of smoking during pregnancy and the postpartum period: an umbrella review
Source: BMC Pregnancy Childbirth. 2021 Mar 26;21:254. doi: 10.1186/s12884-021-03729-1 (PMC7995767; doi:10.1186/s12884-021-03729-1)
Supplement: Supplementary file 1 — Additional file 1. MEDLINE Search strategy and Tables S1-S10. [file 12884_2021_3729_MOESM1_ESM.docx]

Contents

[MEDLINE search strategy 2](#_Toc49847575)

[Additional file Table 1: Data extraction tool 3](#_Toc49847576)

[Additional file Table 2: Characteristics of included reviews 4](#_Toc49847577)

[Additional file Table 3: Impacts of smoking during pregnancy on mothers 12](#_Toc49847578)

[Additional file Table 4: Impacts of SDP on infants: results of the meta-analyses 13](#_Toc49847579)

[Additional file Table 5: Impacts of SDP on infants: results of the studies with no meta-analysis 17](#_Toc49847580)

[Additional file Table 6: Dose-response association between SDP and health conditions 18](#_Toc49847581)

[Additional file Table 7: Impact of postnatal maternal smoking on the infants 20](#_Toc49847582)

[Additional file Table 8: Impact of partner’s smoking 21](#_Toc49847583)

[Additional file Table 9: Subgroup analyses: impact of adjustments on results 23](#_Toc49847584)

[Additional file Table 10: Subgroup analyses: impact of study quality on results 25](#_Toc49847585)

[References 27](#_Toc49847586)

# MEDLINE search strategy

1. pregnan*.mp.
2. exp Pregnancy/
3. postpartum.mp. or exp Postpartum Period/
4. 1 or 2 or 3
5. exp Smoking/ or smok*.mp.
6. exp Tobacco/ or tobacco.mp.
7. 5 or 6
8. 4 and 7
9. pregnancy outcomes.mp. or exp Pregnancy Outcome/
10. birth outcomes.mp. or exp Infant, Newborn/
11. exp Child Development/ or childhood outcomes.mp.
12. exp Risk Factors/ or adverse outcomes.mp.
13. "quality of life".mp. or exp "Quality of Life"/
14. "long-term outcomes".mp. or exp Follow-Up Studies/
15. 9 or 1 0 or 11 or 12 or 13 or 14
16. 8 and 15
17. systematic review.mp. or exp "systematic review"/
18. meta-analysis.mp. or exp Meta-Analysis/
19. 18 or 19
20. 16 and 19
21 . limit 20 to (English language and humans)

# Additional file Table 1: Data extraction tool

| Date |  |
| --- | --- |
| First Author & Publication Year |  |
| Study Focus |  |
| Mother or Infant Investigated (M/I) |  |
| Study design |  |
| Years Included |  |
| Databases Searched |  |
| Any Limitations to Database Search |  |
| Setting/Country of Included Studies |  |
| Number of Included Studies |  |
| Number of participants |  |
| Main Outcomes |  |
| Heterogeneity |  |
| Risk of bias assessment |  |
| Quality Assessment Tool |  |
| Method of analysis |  |
| Dose-response Test |  |
| Partner Inclusion |  |
| Number of Exposed |  |
| Number of Unexposed |  |
| Number Affected |  |
| Number of Not Affected |  |
| Sub-group analyses (biochemical verification, study quality, etc.) | |

# Additional file Table 2: Characteristics of included reviews

| First Author & Year | Meta-analysis | Focus | M/I | Years Included | Databases Searched | Any Limitation | Countries | Quality Score |
| --- | --- | --- | --- | --- | --- | --- | --- | --- |
| Ananth 1999^1^ | Yes | Placental  abruption & SDP | M | 1966 to 1997 | MEDLINE | English only | Mostly HICs | **8** |
| Ankum 1996^2^ | Yes | Risk factors for ectopic pregnancy | M | 1978 to 1994 | MEDLINE | Studies in English, French, German, Dutch | Not provided | **7** |
| Antonopoulos 2011^3^ | Yes | Lympoma & SDP | I | All by Aug. 2010 | Medline, Embase, Scopus, Google Scholar, Ovid and the Cochrane Library | Not Specified | HICs | **12** |
| Brion 2008^4^ | Yes | Blood pressure & SDP | I | Not provided | Not provided | Studies were included if the sample size > 100 | Not provided | **7** |
| Burke 2012^5^ | Yes | Asthma and wheezing & SDP and passive smoking | I | 1997 to Feb. 2011 | Medline, Embase, Cumulative Index to Nursing and Allied Health Literature, AMED, and conference abstracts | Restricted to children (0-18), prospective cohort studies only, only English studies, studies ascertained smoking status at the same time as disease outcome were not included | Mostly HICs | **13** |
| Castles 1999^6^ | Yes | Pregnancy complications & SDP | M | 1966 to May 1995 | MEDLINE and Current Contents | English studies only | HICs | **7** |
| Chao 2014^7^ | No | Risks for tourette syndrome | I | All by October 2012 | Medline, Embase and PsycINFO | English and French studies | Not provided | **7** |
| Chrestani 2013^8^ | No | Risks for accelerated growth in childhood | I | Not stated | PubMed/ MEDLINE | Children aged between 0 and 12 years, studies in English, Portuguese or Spanish, studies including "catch-up" as an outcome | HICs | **8** |
| Chu 2016^9^ | Yes | Childhood neuroblastoma & SDP | I | 1946 to Jul. 2014 | PubMed, EMBASE, Cochrane Library, and ISI Web of Science | English studies only | HICs | **12** |
| Clifford 2012^10^ | No | Cognitive parameters of children and young adults & SDP | I | Jan. 2000 to Dec. 2011 | Medline, Science Direct, Google Scholar, CINAHL, EMBASE, Zetoc and Clinicaltrials.gov | Passive smoking by pregnant women who were non-smokers was not included | HICs | **6** |
| Conde-Agudelo 1999^11^ | Yes | Preeclampsia & SDP | M | 1966 to 1998 | MEDLINE, Embase, Popline, CINAHL, Lilacs | The definition of preeclampsia corresponded to hypertension after 20 weeks’ gestation plus proteinuria | Mostly HICs | **13** |
| DiFranza 1995 | Yes | Sudden infant death & SDP | I | All by 1995 | Microfilm archives at their University. | Papers not included if predated microfilm archives at their University. English only. | HICs | **6** |
| England 2007^12^ | No | Preeclampsia & SDP | M | All by Mar. 2006 | MEDLINE | English only | HICs | **7** |
| Faiz 2003^13^ | Yes | Placenta previa & SDP | M | Jan. 1966 to Mar.2000 | MEDLINE | English only | Mostly HICs | **12** |
| Fernandes 2015^14^ | No | Vision difficulties & SDP | I | 1985 to 2013 | MEDLINE, EMBASE, Global Health, PsycINFO, Scopus, Web of Science and Google Scholar | Studies assessing children aged 0–18 years, Studies with comparable groups | Mostly HICs | **10** |
| Ferrante 2014^15^ | No | Asthma & smoke exposure | I | Jan. 2010 to  Dec. 2013 | PubMed and Scopus | Not Specified | Not provided | **6** |
| Flenady 2011^16^ | Yes | Risks for stillbirth | I | 1998 to 2009 | Medline (PubMed and Ovid), CINAHL, and the Cochrane Database of Systematic Reviews | English studies only, stillbirth definition of “20 weeks’ gestation or more or a birthweight of min. 400gr.” | HICs | **11** |
| Hackshaw 2011^17^ | Yes | Birth defects & SDP | I | 1959 to 2010 | MEDLINE | Only English studies | Mostly HICs | **12** |
| Huang 2007^18^ | No | Oveweight or obesity & prenatal factors | I | Jan. 1975-Dec.2005 | PubMed and MDConsult | Only English studies including children aged between 5 and 21 | Not provided | **5** |
| Huang 2014^19^ | Yes | Brain tumors & SDP | I | All by Jan.2014 | PubMed and Embase | Not Specified | Mostly HICs | **14** |
| Huang 2016^20^ | Yes | Risks for non-genetic intellectual disability | I | All by Sept. 2015 | Medline and Embase | Studies providing min. information to estimate RRs were included. | HICs | **11** |
| Huncharek 2002^21^ | Yes | Brain tumors & SDP | I | Jan. 1966 to 2001 | MEDLARS, EMBASE, CancerLit | Pediatric patients only | Not provided | **11** |
| Ino 2010^22^ | Yes | Obesity & SDP | I | Jan. 2000 to Ap. 2008 | PubMEd | English only | HICs | **9** |
| Jayes 2016^23^ | Yes | Respiratory health & smoking | M & I | All by 2013 | MEDLINE, Embase, and Web of Science | Not Specified | Mostly HICs | **13** |
| Jenabi 2017^24^ | Yes | Hypremasis gravidarum & SDP | M | All by Jan. 2016 | PubMed, Web of Science, and Scopus | No limitation | Mostly HICs | **10** |
| Jones 2011^25^ | Yes | Lower respiratory infections & household smoking | I | 1997 to Nov. 2010 | MEDLINE and EMBASE | Comparative epidemiological studies, no language restriction during the search but reported English studies only | Mostly HICs | **14** |
| Jones 2012^26^ | Yes | Middle ear disease & parental smoking | I | Jan. 1997 to Dec. 2010 | MEDLINE, EMBASE, and CAB abstracts | Only English studies were reported | Mostly HICs | **13** |
| Kantor 2016^27^ | Yes | Atopic Dermatitis and smoke exposure | I | 1823 to 2015 | MEDLINE, EMBASE, Scopus, and Cochrane Library | Not Specified | Not provided | **12** |
| Lancaster 2010^28^ | No | Risk factors for depressive symptoms | M | Jan. 1980 to Mar. 2008 | PubMed, CINAHL, SCOPUS, PsycINFO, Sociological Abstracts, ISI Proceedings, ProQuest Dissertations &Theses. | English only, studies with more than 20 subjects were included | HICs | **9** |
| Latimer 2012^29^ | No | Risks for disruptive behaviour disorders | I | Jan. 1966 and 31 Ap. 2009 | PsycLIT/PsycInfo, BNI,  CINAHL, EBM, EMBASE, ERIC, MEDLINE, Social Science Citation Index, and Science Citation Index. | Studies including assessment of factors present antenatally and in the first four years of life | Not provided | **7** |
| Lee 2013^30^ | Yes | Congenital heart defects & SDP | I | 1947 to July 2011 | Medline (Ovid and PubMed) | English studies only | Mostly HICs | **10** |
| Linnet 2003^31^ | No | Maternal risk factors for attention deficit hyperactivity disorder | I | 1966 to January 2002 | PubMed, MEDLINE, EMBASE, and PsycINFO | Children aged four years and older | HICs | **6** |
| Little 2004^32^ | Yes | Oral clefts & SDP | I | 1966 to 2002 | EMBASE and MEDLINE | Not Specified | Not provided | **9** |
| Marufu 2015^33^ | Yes | Stillbirth & SDP | I | All by 31 December 2012 | MEDLINE, EMBASE, Psych Info and Web of Science | Stillbirth was defined as "fetal loss or death at 20 weeks gestation and above" | Mostly HICs | **13** |
| Mitchell 2006^34^ | Yes | SIDS & SDP | I | Not provided | MEDLINE and EMBASE | Not Specified | Mostly HICs | **6** |
| Nicoletti 2011^35^ | Yes | Birth defects & SDP | I | 1950 to Ap. 2010 | MEDLINE, LILACS and SciELO | Not Specified | Mostly HICs | **14** |
| Oken 2008^36^ | Yes | Overweight & SDP | I | 1966 to Jun. 2006 | Medline | Children older than two were included, studies used only a continuous measure of adiposity were not included in the meta-analysis, no language limit was defined but selected studies were all in English | HICs | **10** |
| Park 2008^37^ | Yes | Inrtauterine tobacco exposure & breast cancer risk in the females | I | Jan. 1966 to Feb.2007 | MEDLINE | English only | HICs | **9** |
| Pereira 2017^38^ | Yes | LBW & SDP | I | 1984 to 2016 | Medline, Embase, LILACS, SciELO, Web of Science, and Scopus | Not Specified | Mostly UMICs | **13** |
| Pineles 2014^39^ | Yes | Miscarriage & SDP | M | 1956 to Aug. 2011 | PubMed | Only studies  comparing women who  were exposed and those who were not exposed to tobacco  smoke from cigarettes included | Mostly HICs | **11** |
| Pineles 2016^40^ | Yes | Perinatal death (stillbirth and neonatal death) & SDP | I | 1956 and Aug. 31, 2011 | PubMed | Not Specified | Mostly HICs | **12** |
| Rayfield 2017^41^ |  | Childhood overweight and obesity & SDP | I | All by Jan. 2015 | PubMed, EMBASE, Global Health and Cinahl and Web of Science | Children born at full term (>37 weeks), overweight measured between 2 and 18 years | Mostly HICs | **14** |
| Riedel 2014^42^ | Yes | Childhood obesity & SDP | I | 1900 to 2013 | MEDLINE, EMBASE and Web of Knowledge | English only | Mostly HICs | **11** |
| Rosen 2014^43^ | Yes | Autism & SDP | I | All by Dec. 2013 | Pub Med and Google Scholar | Prospective studies only | HICs | **12** |
| Rumrich 2016^44^ | Yes | Childhood cancers & SDP | I | All by 1 Jun. 2015 | PubMed and Web of Science | Cancer patients younger than 20 | Mostly HICs | **11** |
| Shah 2000^45^ | Yes | Preterm delivery & SDP | I | 1966 to 1997 | MEDLINE | English only, prospective studies only | Mostly HICs | **8** |
| Shobeiri 2017^46^ | Yes | Placenta previa & SDP | M | All by Jun. 2015 | PubMed, Web of Science ans Scopus | Not Specified | Mostly HICs | **11** |
| Shobeiri 2017a^47^ | Yes | Placenta abruption & SDP | M | All by Ap.2016 | PubMed, Web of Science ans Scopus | Not Specified | Mostly HICs | **11** |
| Tang 2015^48^ | Yes | Autism & SDP | I | All by 10 Jun. 2015 | PubMed, EMBASE, Web of Science, and Cochrane Library | Not Specified | HICs | **10** |
| Tsai 2017^49^ | No | Health impacts of environmental pollutants | I | All by Jan 2017 | PubMed | Only studies with a birth cohort from Asian countries and published in English | UMICs | **4** |
| Tripathee 2016^50^ | Yes | Risks for microtia | I | January 2000 to Oct. 2014 | MEDLINE, EMBASE, and Google scholar | Studies without a control group were not included, English only | Mostly HICs | **8** |
| Tuomisto 2009^51^ | Yes | Testicular cancer in son & SDP | I | All by January 2008 | PubMed | Not Specified | Not provided | **8** |
| Wang 2014^52^ | Yes | Neural tube defects & SDP | I | Jan. 1, 1957 to Aug.31, 2012 | PubMed, MEDLINE, Elsevier Science, and Springer Link | English studies only | Mostly HICs | **11** |
| Waylen 2009^53^ | Yes | Assisted reproduction & SDP | I | 1806 to May 2007 | Medline, Embase, Cochrane library, PsycINFO, Science Citation Index, Social Science Citation Index , CINAHL | Studies focussing on assisted reproduction were included, English only | Not provided | **10** |
| Wei 2015^54^ | Yes | Preeclampsia & SDP | M | All by Aug. 2015 | PubMed and Web of Science | No information provided | HICs | **11** |
| Wendland 2008^55^ | Yes | Gestational diabetes & SDP | M | All by 2007 | PubMed, Embase, LILACS and CINAHL | Excluded Type 1 diabetes studies | HICs | **14** |
| Weng 2012^56^ | Yes | Overweight & SDP | I | 1990 to May 2011 | MEDLINE, EMBASE, PubMed and CAB Abstracts | Children aged between two and 16, prospective studies only with at least two-year follow up | Mostly HICs | **12** |
| Wyszynski 1997^57^ | Yes | Oral clefts & SDP | I | Jan.1966 to Apr.1996 | MEDLINE and Current Contents | Only the studies using oral clefts as cases were included. | HICs | **9** |
| Xuan 2016^58^ | Yes | Oral clefts & SDP | I | Jan.1966 to May 2015 | Cochrane, PubMed, and Ovid Medline | Language restriction was not define but all selected studies are in English, Studies with precise information on maternal smoking were included | Not provided | **10** |
| Yan 2015^59^ | Yes | Childhood acute lymphoblastic leukemia | I | Not Specified | Medline, PubMed, and Web of Science | Children younger than 15, case-control studies, English only | HICs | **12** |
| Yermachenko 2015^60^ | Yes | Menarche age & SDP | I | 1995 to 2014 | PubMed, EMBASE, CINAHL, Web of Science, and Google Scholar | English, French, Russian studies | HICs | **11** |
| Zhang 2017^61^ | Yes | Congenital heart diseases & SDP | I | All by 24.07.2015 | PubMed, EMBASE, and Web of Science | English only | Mostly HICs | **10** |
| Zhang 2013^62^ | Yes | SIDS & SDP | I | 1990 to 2011 | PubMed and CNKI (China National Knowledge Infrastructure) | 6-criteria-item developed by the American Academy of Pediatrics Task Force was applied | Mostly HICs | **10** |
| Zwink 2011^63^ | Yes | Risks for anorectal malformations (ARMs) | I | All by August 2010 | PubMed, EMBASE, ISI Web of Knowledge and the Cochrane Library | English only, studies reporting 2 or more ARM outcomes | HICs | **9** |

# Additional file Table 3: Impacts of smoking during pregnancy on mothers

| Health Condition | Effect of SDP (OR/RR, CI, number of studies) | Heterogeneity | First Author & Year |
| --- | --- | --- | --- |
| Abruptio placenta | OR= 1.9 (95% CI, 1.8-2.0, n=13)  OR=1.62 (95% CI 1.46- 1.77, n=8)  OR= 1.80 (95% CI, 1.75-1.85, n=27) | Q=40.9, P=0.001  Not Provided  I²=78.1%, P=0.000 | Ananth 1999^1^  Castles 1999^6^  Shobeiri 2017a^47^ |
| Asthma exacerbations | Number of exacerbations increased in smoker or ex-smoker pregnant women in one study. | NA | Jayes 2017^23^ |
| Depressive symptoms during pregnancy | Based on 11 studies the author found no significant impact of SDP on depressive symptoms. | NA | Lancaster 2010^28^ |
| Ectopic pregnancy | **Case control studies with pregnant controls** OR=2.3 (95% CI, 2.0-2.8, n=6)  **Case control studies with non-pregnant controls** OR= 1.6 (95% CI, 1.4 - 2.0, n=2)    OR=1.77 (95% CI, 1.31-2.22, n=9)  **In assisted reproduction** OR= 15.69 (95% CI 2.87-85.76, n=3) | P=0.56, P=0.50  P=0.50, P=0.25  Not Provided  Not Provided | Ankum 1996^2^  Castles 1999^6^  Waylen 2009^53^ |
| Hyperemasis gravidarum | OR= 0.40 (95% CI, 0.24-0.56, n=11) | P=0.000, I²= 93.5% | Jenabi 2017^24^ |
| Miscarriage | RR= 1.32 (95% CI, 1.21-1.44; n= 25 studies) | P < 0.01 | Pineles 2014^39^ |
| Placenta previa | OR=1.58 (95% CI, 1.04-2.12, n=6)  OR= 1.42 (95% CI, 1.30-1.54, n=21) | Not Provided  I²= 62.7%, P=0.000 | Castles 1999^6^  Shobeiri 2017^46^ |
| PPROM | OR=1.7 (95% CI, 1.18-2.25, n=6) | Not Provided | Castles 1999^6^ |
| Preeclampsia | OR= 0.51 (95% CI, 0.38-0.64,n=5)  OR= 0.68 (95% CI, 0.57-0.81, n=27)  Studies reported up to 50% decrease in the risk.  RR= 0.67 (95% CI, 0.60-0.75, n=17) | Not Provided  Q=5.9; P=0.45  NA  P<0.001 I²=91.7% | Castles 1999 Conde-Agudelo 1999 ^11^ England 2007^12^  Wei 2015^54^ |
| Spontaneous miscarriage in assisted reproduction | OR=2.65 (95% CI, 1.33-5.30, n=7) | P=0.006, I²=45.4% | Waylen 2009^53^ |

# Additional file Table 4: Impacts of SDP on infants: results of the meta-analyses

| Health Condition | Effect of SDP (OR/RR, CI, number of studies) | Heterogeneity | First Author & Year |
| --- | --- | --- | --- |
| Asthma | **In children who are 2 & younger,** OR = 1.85 (95% CI, 1.35–2.53, n=5) **In children aged 3 and 4** **years**  OR=1.30 (95% CI, 0.88–1.92, n=1) **In children aged 5 to 18 years** OR=1.23 (95% CI, 1.12–1.36, n=8) | ≤2; I²=41.9%, 5-18; I²= 50% | Burke 2012^5^ |
| Atopic dermatitis | OR=0.94 (95% CI, 0.68-1.10, n=UNK) | P<0.0001, I²=74.7% | Kantor 2016^27^ |
| Autism | OR=1.02 (95% CI, 0.93–1.12, n=15)  OR=1.02 (95% CI, 0.93-1.13, n=15) | I²=81%  p< 0.01, I²=67.3% | Rosen 2014^43^  Tang 2015^48^ |
| Birth defects | **Any type of defect** OR= 1.18 (95%CI, 1.14-1.22, n=188) **Cardiovascular system** OR= 1.11 (95% CI, 1.03-1.19, n=29) **Digestive system** OR= 1.18 (95% CI, 1.07-1.30,n=22) **Musculoskeletal system** OR= 1.27 (95% CI, 1.16-1.39, n=48) **Face and neck** OR= 1.28 (95% CI, 1.19-1.37, n=53) **Respiratory system** OR=1.11 (95% CI, 0.93-1.32, n=6)  **Uregenital system** OR= 1.04 (95% CI, 0.94-1.12, n=45) **Nervous system** OR=1.09 (95% CI, 0.98-1.21, n=35)  **Any type of defect** OR= 1.01 (95% CI, 0.96-1.07, n=38)  **Cardiovascular defects** OR=1.09 (95% CI, 1.02 –1.17, n=25) **Musculoskeletal defects** OR=1.16 (95% CI, 1.05– 1.27, n=25) **Craniosynostosis** OR=1.33 (95% CI, 1.03 –1.73, n=5) **Facial defects** OR=1.19 (95% CI, 1.06 –1.35, n=12) **Eye defects** OR=1.25 (95% CI, 1.11 –1.40, n=8) **Oral clefts** OR=1.28 (95% CI, 1.20 –1.36, n=38) **Gastrointestinal defects** OR=1.27 (95% CI, 1.18 –1.36, n=35) **Genitourinary**  OR=1.05 (95% CI, 0.98-1.12, n=40) **Central nervous system** OR= 1.10 (95% CI, 1.01-1.19, n=29) **Respiratory system** OR= 1.11 (95% CI, 0.95-1.30, n=6)  **Skin defects** OR=0.82 (95% CI, 0.75– 0.89, n=5) | **Any type** I²=77.2% **CV system** I² = 58.7% **Digestive system** I² = 21.7%  **Musc. system** I² = 78.5 **Face and neck** I² = 53.7% **Respiratory system** I² = 0.00% **Uregenital system** I² = 66.8% **Nervous system** I² = 53.5 %  **Any type** p<0.00001, I²=79%  **CV. Defects** p<0.00001, I²= 64% **Musculosketal**  p<0.00001, I² =90% **Craniosynostosis** P=0.09, I²=51% **Facial**  P<0.00001, I²=77% **Eye**  p=0.002, I²=57%  **Oral** p=0.003, I²=43% **Gastrointestinal** p=0.02, I² =36%  **Genitourinary**  P<0.00001, I²=61%  **CNS** p= 0.02, I²=49% **Respiratory** p=0.18, I² =0%  **Skin** p= 0.00001, I²=0% | Nicoletti 2014^35^  Hackshaw 2011^17^ |
| Birth defects  (continued) | **Anorectal malformations** OR=1.03 (95% CI, 0.83-1.29, n=6)  **Congenital heart defects *Overall*** RR= 1.11 (95% CI, 1.02-1.21, n=19) ***Septal defects*** RR=1.44 (95% CI, 1.16-1.79, n=6)  **Congenital heart defects** RR= 1.11 (95% CI,1.04, 1.18)  **Neural tube defects**  ***All NTDs*** OR= 1.39 (95% CI, 1.18-1.64, n=3) ***Spina Bifida***  OR=1.55 (95% CI, 1.06-2.26, n=13)  **Oral clefts**  ***All clefts*** RR=1.34 (95% CI, 1.25-1.44, n=15) ***Cleft palate***  RR=1.22 (95% CI, 1.10-1.35, n=12)  **Cleft lips with or without cleft palate (CL/P)** OR= 1.29 (95% CI, 1.18-1.42, n=11) **Cleft Palate (CP)** OR= 1.32 (95% CI,1.10-1.62,n=9)  **All clefts** OR=1.40 (95% CI,1.26-1.49, n=11) **Cleft palate only** OR=1.24 (95% CI 1.12-1.38, n=19) | p=0.12, I²=42.6%    p<0.001  P=0.001  p<0.001, I²=69%  P=0.003, I²=65.8%  p<0.000, I²=73.2 %  **All clefts** P=0.003 **Cleft Palates**  P=0.28  **CL/P** χ2=19.79, P= 0.03, **CP**  χ2= 11.26, P=0.20  **All clefts** I²=53.1%, P=0.019  **Cleft Palates** I²=35.1%, P=0.066 | Zwink 2011^63^  Lee 2013^30^  Zhang 2017^61^  Wang 2014^52^  Little 2004^32^  Wyszynski 1997^57^  Xuan 2016^58^ |
| Blood Pressure | 0.62 mm Hg, (95% CI, 0.19-1.05, n=7) | p=0.308, I²= 16.4% | Brion 2010^4^ |
| Bone cancer | OR= 0.79 (95% CI, 0.50-1.26, n=2) | I²=0.2 | Rumrich 2016^44^ |
| Brain tumors | OR=1.05 (95% CI, 0.90–1.21, n=12) | p>0.5 | Huncharek 2002^21^ |
| Breast cancer in daughters | OR= 0.98 (95% CI, 0.86-1.13, n=9) | p=0.06 | Park 2008^37^ |
| Central nervous system tumors | OR = 1.09 (95% CI, 1.02–1.17, n=22) | p=0.1, I²=0.3% | Rumrich 2016^44^ |
| Germ cell cancer | OR= 0.92 (95% CI, 0.68-1.24, n=4) | I²=UNK | Rumrich 2016^44^ |
| Gestational diabetes in daughters | OR= 1.03 (99% CI, 0.85-1.25, n=12) | p<0.01 | Wenland 2008^55^ |
| Hepatic tumors | OR= 0.97 (95% CI, 0.70, 1.36, n=3) | I²<0 | Rumrich 2016^44^ |
| Intellectual disability | OR=1.10 (95% CI, 1.06-1.15, n= 5) | p <0.001, I²=0.53 | Huang 2016^20^ |
| LBW | Overall: OR= 2 (1.77-2.26, n=30)  North America:  OR= 1.75 (95% CI, 1.42–2.10, n=7) | p=0.000, I²=66%  p= 0.013, I²=63% | Pereira 2017^38^ |
| Leukemia | **Childhood leukemia**  OR= 0.92 (95% CI, 0.82-1.3, n=21)  **Childhood acute lymphoblastic leukemia**  OR= 0.99 (95% CI, 0.92-1.06, n=19)  **Childhood acute lymphoblastic leukemia**  OR= 1.09 (95% CI, 1.01-1.18, n=9) | I²=0.5  I²=0.6  P=0.981 | Rumrich 2016^44^  Yan 2015^59^ |
| Live birth per cycle in assisted reproduction | OR=0.54 (95% CI, 0.30–0.99, n=18) | I²=UNK | Waylen 2009^53^ |
| Lower respiratory infections (LRI) | RR=1.19 (95% CI, 1.10-1.29, n=11)  OR=1.24 (95% CI, 1.11-1.38, n=10) | I²=78%  I²=77% | Jayes 2016^23^  Jones 2011^25^ |
| Lymphoma, hodgkin lymphoma & non-hodgkin lymphoma | **Lymphoma**  OR= 1.21 (95% CI, 1.05-1.34, n=6)  **Lymphoma**  OR=1.10 (95% CI, 0.96–1.27, n=8)  **Hodgkin lymphoma**  OR= 0.90 (95% CI, 0.66–1.21, n=4)  **non-Hodgkin lymphoma**  OR= 1.22 (95% CI, 1.03–1.45, n=8) | p=0.9, I²<0  **L**  P=0.54, I²=0.0%  **HL**  p= 0.91, I²=0.0%  **NHL**  P=0.41, I²=2.7% | Rumrich 2016^44^  Antonopoulos 2011^3^ |
| Microtia | OR= 1.05 (95% CI, 0.63-1.77, n=3) | P= 0.84, I²=68% | Tripathee 2016^50^ |
| Middle ear disease | OR= 1.11 (95% CI, 0.93-1.31, n=6) | I²=62% | Jones 2012^26^ |
| Neuroblastoma | OR= 1.30 (95% CI, 1.10-1.53, n=5) | I²=<0 | Rumrich 2016^44^ |
| Obesity | OR=1.52 (95%CI,1.36–1.70, n=16)  OR= 1.60 (95% CI, 1.37-1.88, n=4)  OR=1.55 (95% CI, 1.40-1.73, n=16) | p<0.0001  I²=32.47%  I²=24% | Ino 2010^22^  Riedel 2014^42^  Rayfield 2017^41^ |
| Overweight | OR= 1.50 (95% CI, 1.36- 1.65, n=14)  OR=1.47 (95% CI, 1.26-1.73, n=7)  OR= 1.33 (95% CI, 1.23-1.44, n=8)  OR= 1.37 (95% CI, 1.28 to 1.46, n=31) | p=0.02, I²=49%  p=0.064, I²=47.5%  I²=0.00%  I²=45% | Oken 2008^36^  Weng 2012^56^  Riedel 2014^42^  Rayfield 2017^41^ |
| Perinatal death & neonatal death | **Perinatal death** RR= 1.33 (95% CI, 1.25-1.41, n= 46) **Neonatal death** RR= 1.22 (95% CI,1.14-1.30, n= 28) | **Perinatal death** I² = 60%, P<0.0001  **Neonatal death** I² =39%, P<0.05 | Pineles 2016^40^ |
| Preterm birth | OR= 1.27 (95% CI, 1.21-1.33, n=20) | Analysed qualitatively | Shah 2000^45^ |
| Reduced menarche age | RR= 1.15 (95% CI, 1.07-1.23, n=3) | p= 0.003, I²=58% | Yermachenko 2015^60^ |
| Renal tumors | OR= 0.95 (95% CI, 0.75-1.22, n=3) | I²<0 | Rumrich 2016^44^ |
| SIDS | OR= 1.32 (1.18-1.48, n=23)  RR= 2.86 (95% CI, 2.77-2.95, n=47)  OR= 2.25 (95% CI, 2.03-2.50, n=23) | p<0.001 χ2=36.22 p=0.87  p<0.001, I²=76.6% | DiFranza 1995  Mitchell 2006^34^  Zhang 2013^62^ |
| Soft tissue tumor | OR= 0.89 (95% CI, 0.71- 1.12, n=5) | I²<0 | Rumrich 2016^44^ |
| Stillbirth | OR= 1.36 (95% CI, 1.27-1.46, n=4)  OR=1.55 (95% CI, 1.36-1.78, n=11)  RR = 1.46 (95% CI, 1.38-1.54, n = 57) | p=0.368, I²=4.9%  I²=79%  I²=67%, P < 0.0001 | Flenady 2011^16^  Marufu 2015^33^  Pineles 2016^40^ |
| Testicular cancer in sons | OR= 1.0 (95% CI, 0.88-1.12, n=7) | p=0.80 | Tuomisto 2009^51^ |
| Wheezing | **In children 2 years old or younger** OR= 1.41 (95% CI, 1.20-1.67, n=14) **In children aged 3 and 4 years** OR=1.28 (95% CI, 1.14-1.44, n=8) **In children aged 5 to 18 years** OR= 1.52 (95% CI, 1.23-1.87, n=5) | ≤2; I² =82.5%    3-4; I²=65.5% 5-18; I²=21.1% | Burke 2012^5^ |

# Additional file Table 5: Impacts of SDP on infants: results of the studies with no meta-analysis

| Health Condition | Effect of SDP (main outcome) | First Author & Year |
| --- | --- | --- |
| Accelerated growth in childhood | Studies reported that children of smoking mother gained weight faster than children of non-smokers. | Chrestani 2013 |
| Asthma | Studies showed a positive association between SDP and asthma. | Ferrante 2014 |
| Cognitive parameters of children and young adults | **Poor school performance**  ***1–9 cigarettes per day***  OR= 1.59 (95% CI, 1.55-1.63, n=1);  ***≥ 10 cigarettes per day*** OR=1.92 (95% CI, 1.86-1.98, n=1)  0.26 point yearly reduction in Peabody Individual Achievement Test was observed in one study.  Decreased IQ was observed in two studies.  **Intellectual impairment**  aOR= 1.22 (95% CI, 1.14-1.31, n=1)  aRR=1.12 (95% CI, 0.92-1.36, n=1)  **Neurodevelopment**  2.5 point deficit (95% CI,− 6.3-1.2, P=0.18) | Clifford 2012 |
| Lung function in childhood | Current evidence is not sufficient to suggest a significant association between SDP and lung function in children. | Jayes 2016 |
| Disruptive behaviour disorders | ***Attention deficit hyperactivity disorder***  OR=2.1 (95% CI, 1.1-4.1, n=1)  OR= 2.7 (95% CI, 1.1-7.0, n=1)  OR= 4.4 (95% CI, 1.2-15.5, n=1)  RR= 2.5 (95% CI, 0.8-7.3, n=1)  15 studies showed a relationship between SDP and increased incidence of ADHD in the child. | Linnet 2003  Latimer 2012 |
| Overweight and obesity | Eight studies reported positive association, ORs ranging between 1.1 and 2.9. | Huang 2006 |
| Sleep apnea | In infants (1 day-29 weeks) RR= 1.76 (95% CI, 1.17-2.64, n=1) | Jayes 2016 |
| Tourette syndrome (TS) & attention deficit disorder | **TS vs. control** OR = 4.6 (95% CI, 0.45-46.6, n=1) **TS& ADHD vs. control** OR = 8.5 (95% CI, 0.97-75.2, n=1) **TS& ADHD vs. TS** OR = 2.43 (95% CI, 1.23-4.82, n=1) **Presence of OCD** OR = 8.27 (95% CI, 0.87-78.20, n=1) | Chao 2014 |
| Vision difficulties | SDP is associated to several vision problems according to 18 studies. | Fernandes 2015 |

# Additional file Table 6: Dose-response association between SDP and health conditions

| Condition | First threshold | | Second and third thresholds | First Author & Year |
| --- | --- | --- | --- | --- |
| Birth defects | ***9 or less cig/day***  OR=1.19 (95% CI, 1.12-1.26, n=60, I²=UNK) | | ***10-19 cig/day***  OR= 1.29 (95% CI, 1.20-1.38, n=60, I²=UNK)  ***20 or more cig/day***  OR= 1.41 (95% CI, 1.30-1.52, n=60, I²=UNK) | Nicoletti 2014 |
| Central nervous system tumors | ***10 or less cig/day***  OR= 1.06 (95% CI, 0.92-1.21, n=14, I²=0.4%) | | ***11-20 cig/day***  OR= 1.07 (0.90, 1.27, n=8, I²<0)  ***21 or more cig/day***  1.01 (95% CI, 0.71-1.44, n=6, I²<0) | Rumrich 2016 |
| Childhood acute lymphoblastic leukemia | ***1–10 cigarettes/day vs 11 or more***  OR=1.03 (95% CI, 0.74-1.42, n=UNK) | | ***1–20 cigarettes/day vs 21 or more***  OR=1.11(95% CI, 0.70-1.73, n=UNK) | Yan 2015 |
| Congenital heart defects | ***Light smokers***  RR= 0.99 (95% CI, 0.92–1.06, n=UNK) | | ***Heavy smokers***  RR=1.04 (95% CI, 0.86–1.26, n=UNK) | Lee 2013 |
| Lymphoma | ***Increased risk per cigarette***  0.02 ( 95%, CI, -0.018- 0.06 n=UNK, p=0.29)  ***10 or less cig/day***  OR= 1.28 (95% CI, 0.96-1.69, n=3, I²<0) | | ***NA***  ***11 or more cig/day***  OR=1.19 (95% CI, 0.80-1.78, n=2, I²<0) | Antonopoulos 2011  Rumrich 2016 |
| Menarche age | ***9 or less cig/day***  Std dif in means  -0.094 (95%CI, -0.245-0.57, n=5, I² = 71%) | | ***10-19 cig/day***  Std dif in means  -0.080 (0.232- 0.072,n= 5I² =61%)  ***20 or more cig/day***  Std dif in means  0.140 (-0.172-0.453, n=3, I² =85%) | Yermachenko 2015 |
| Miscarriage | ***10 or less cig/day***  RR= 1.08 (95% CI, 0.96-1.21, n= 16, I²=UNK) | | ***11–19* cig/day** RR=1.25(95% CI,1.17-1.34, n = 9, I²=UNK)  ***20 or more* cig/day** RR= 1.42 (95% CI,1.19-1.70, n = 11, I²=UNK) | Pineles 2014 |
| Neonatal death | ***10 or less cig/day***  RR= 1.06 (95% CI, 0.90-1.26, n=10, I²=UNK) | | ***11-20 cig/day***  RR= 1.30 (95% CI, 1.00-1.68, n=4, I²=UNK)  **21 or more cig/day**  RR= 1.31 (95% CI, 1.11-1.55, n=5, I²=UNK) | Pineles 2016 |
| Neuroblastoma | ***9 or less cig/day***  OR= 0.97 (95% CI, 0.62-1.53, n=3, I²= 58%) | | ***10 or more cig/ day***  OR= 1.20 (95% CI, 0.80-1.66, n=3, I²= 48%) | Chu 2016 |
| Perinatal death | | ***10 or less cig/day***  RR= 1.17 (95% CI, 1.05-1.31, n=16, I²=UNK) | ***11-20 cig/day***  RR=1.35 (95% CI, 1.18-1.53, n=6, I²=UNK)  **21 or more cig/day**  RR= 1.40 (95% CI, 1.32-1.50, n=11, I²=UNK) | Pineles 2016 |
| Preeclampsia | | ***9 or less cig/day***  Cohort studies: 0.77 (95% CI, 0.73-0.83, n=9)  Case studies: 0.87 (95% CI, 0.67-1.13, n=3) | ***10 or more cig/day***  Cohort studies:  0.67 (95% CI, 0.63-0.71, n=9)  Case studies:  0.61 (95% CI, 0.47-0.78, n=3) | Conde-Agudelo 1999 |
| Preterm birth | | ***9 or less cig/day***  OR= 1.22 (95% CI, 1.13-1.32, n=6, I²=UNK) | **10-19 cig/day**  OR= 1.38 (95% CI, 1.23-1.55, n=6, I²=UNK)  **20 or more cig/day**  OR= 1.31 (95% CI, 1.20-1.42, n=5, I²=UNK) | Shah 2000 |
| Stillbirth | | ***9 or less cig/day***  OR= 1.09 (95% CI, 0.97-1.24, n=6, I²=0%)    ***10 or less cig/day***  RR= 1.10 (95% CI, 0.98-1.24, n=20, I²=UNK) | ***10 or more cig/day***  OR=1.52 (95% CI, 1.30-1.78, n=7, I²=80)  ***11-20 cig/day***  RR= 1.30 (95% CI, 1.22-1.38, n=9, I²=UNK)  **21 or more cig/day**  RR= 1.24 (95% CI, 1.03-1.50, n=10, I²=UNK) | Marufu 2015  Pineles 2016 |

# Additional file Table 7: Impact of postnatal maternal smoking on the infants

| Health Condition | Main Outcome | Heterogeneity | First Author & Year |
| --- | --- | --- | --- |
| Asthma | ***Aged 2 or younger;***  OR= 2.47 (95% CI, 0.65-9.39, n=2)  ***Aged 3-4;***  OR= 1.05 (95% CI, 0.88-1.25, n=4)  ***Aged 5-18;***  OR= 1.20 (95% CI, 0.98-1.44, n=8) | I²=3.7%  I²=0.0%  I²=65.3% | Burke 2012 |
| Leukaemia | Three months after pregnancy OR= 0.74 (95% CI, 0.52-1.05, n=4) | I²=0.5 | Rumrich 2016 |
| LRI | OR= 1.58 (95% CI, 1.45-1.73, n=31) | I²=76.5% | Jones 2011 |
| Middle ear disease | OR= 1.62 (95% CI, 1.33-1.97, n=20) | I²=93% | Jones 2011b |
| Obesity | Prevalence of obesity OR= 1.6% (95% CI, 0.4%-4.1%, n=1) | NA | Oken 2008 |
| SIDS | OR = 1.97 (95% CI, 1.77-2.19, n=18) | I²=56.4% | Zhang 2013 |
| Wheezing | ***Aged 2 or younger;***  OR=1.70 (95% CI, 1.24–2.35, n=4, I²=0.0%)  ***Aged 3-4;***  OR= 1.65 (95% CI, 1.20–2.28, n=4, I²=48.5%)  ***Aged 5-18;***  OR= 1.18 (95% CI, 0.99–1.40, n=3, I²=1.40%) |  | Burke 2012 |

# Additional file Table 8: Impact of partner’s smoking

| Health Condition | Main Outcome | First Author & Year |
| --- | --- | --- |
| Anorectal malformations | **NOT specific to smoking during pregnancy**  OR=1.53 (95% CI, 1.04-2.26, n=2, P=0.03) | Zwink 2011 |
| Asthma | **NOT specific to smoking during pregnancy**  ***Paternal smoking*** Aged 3-4; OR=1.34 (95% CI,1.23-1.46, n=1) Aged 5-18; OR=0.98 (95% CI, 0.71-1.36, n=3, I²=0.0)  ***Household smoking***  Aged 2 or younger; OR=1.14 (95% CI, 0.94-1.38 n=3 I²=1.7)  Aged 3-4; OR=1.21 (95% CI, 1.00-1.47, n=5, I²=72.7)  Aged 5-18; OR=1.30 (95% CI, 1.04-1.62, n=6, I²=37.7) | Burke 2012 |
| Brain tumors | RR= 0.93 (95% CI, 0.85-1.00, n=9, I²=0%) | Huang 2014 |
| Breast cancer risk in the females | OR= 1.0 (95% CI, 0.9-1.1, n=1) | Park 2008 |
| Delayed mental development | OR= 2.36 (95% CI, 1.21-4.59, n=1) | Tsai 2017 |
| LRTI | **NOT specific to smoking during pregnancy**  ***Paternal smoking***  OR= 1.22 (95% CI, 1.10-1.35, n=21, I²=62%)  ***Household Smoking***  OR= 1.54 (95% CI, 1.40-1.69, n=37, I²=43%) | Jones 2011 |
| Middle ear disease | **NOT specific to smoking during pregnancy**  1.24 (95% CI, 0.98-1.57; n= 12, I²=87%) | Jones 2012 |
| Miscarriage | 1.11 (95% CI, 0.95-1.31, n= 17) | Pineles 2014 |
| Neonatal death | **Any second-hand smoking**  RR=1.37 (95% CI, 1.04-1.82, n=6, I²=UNK) | Pineles 2016 |
| Overweight & obesity | **NOT specific to smoking during pregnancy** ***Paternal smoking***  ***Overweight*** OR=1.07 (95% CI, 1.00-1.16, n=8, I²=0.00%) ***Obesity*** OR= 1.23 (95% CI, 1.10-1.38, n=6, I²=32.47%)  ***Household smoking***  ***Overweight*** OR= 1.22 (95% CI, 1.06-1.39, n=3, I² =72.14%)  ***Obesity*** OR= 1.31 (95% CI, 1.15-1.50, n=3, I² =0.00%) | Riedel 2014 |
| Perinatal death | **Any second-hand smoking**  RR= 1.42 (95% CI, 1.10-1.85, n=2, I²=UNK) | Pineles 2016 |
| Stillbirth | **Any second-hand smoking**  RR=1.37 (95 % CI, 1.04-1.82, n=6, I²=UNK) | Pineles 2016 |
| SIDS | **If mother is non-smoker and father is smoker during pregnancy**  RR= 1.49 (95% CI, 1.29-1.77, n=7, χ2=4.28, p=0.64) | Mitchell 2006 |
| Wheezing | **NOT specific to smoking during pregnancy**  ***Paternal smoking*** Aged 5-18; OR=1.39 (95% CI, 1.05-1.85, n=2, I²=0.0)  ***Household smoking***  Aged 2 or younger; OR=1.35 (95% CI, 1.10-1.64, n=10, I²=64.5)  Aged 3-4; OR=1.06 (95% CI, 0.88-1.27, n=4, I²=54.5)  Aged 5-18; OR=1.32 (95% CI, 1.12-1.56 n=5, I²=1.7) | Burke 2012 |

# Additional file Table 9: Subgroup analyses: impact of adjustments on results

| Health Condition | Unadjusted Ratio | Adjusted Ratio | First Author & Year |
| --- | --- | --- | --- |
| Autism | OR= 1.08 (95% CI, 0.89-1.32, n=6, I²=68.2%) | aOR= 1.00 (95% CI, 0.88-1.13, n=9, I²=69%) | Tang 2015 |
| Birth defects (any type) | OR= 1.19 (95% CI, 1.13-1.25, n=127, I²=76.50%) | aOR= 1.18 (95% CI, 1.13-1.24, n=61, I²=78.80%) | Nicoletti 2014 |
| Central nervous system tumors | OR= 1.17 (95% CI, 0.97, 1.40, n=8, I²= 0.4) | aOR= 1.07 (95% CI, 0.97, 1.19, n=14, I²=0.2) | Rumrich 2016 |
| Gestational diabetes | Unadjusted or adjusted  OR= 1.03 (99% CI, 0.85-1.25, n=12, I²=UNK) | aOR= 0.95 (99% CI, 0.85-1.07, n=5, I²=UNK) | Wenland 2008 |
| Germ cell tumors | Unadjusted or adjusted  OR= 0.92 (95% CI, 0.68-1.24, n=4, I²= 0.5) | aOR= 0.94 (95% CI, 0.66-1.36, n=3, I²=0.7) | Rumrich 2016 |
| Hepatic tumors | OR= 1.04 (95% CI, 0.66-1.63, n=2, I²<0) | Adjusted or unadjusted  aOR= 0.97 (95% CI, 0.70-1.36, n=3, I²<0 ) | Rumrich 2016 |
| Leukaemia | OR= 1.01 (95% CI, 0.85-1.20, n=10, I²=0.5) | aOR= 0.86 (95% CI, 0.74-1.00, n=12, I²=0.5) | Rumrich 2016 |
| Lymphoma | Unadjusted or adjusted  OR= 1.21 (95% CI, 1.01-1.45, n=6, I²<0) | aOR= 1.19 (95% CI, 0.99-1.43, n=5, I²<0) | Rumrich 2016 |
| Miscarriage | RR= 1.27 (95% CI, 1.18-1.37; n=35) | aRR=1.17 (95% CI: 1.04, 1.31; n= 19) | Pineles 2014 |
| Neuroblastoma | OR= 1.42 (95% CI, 0.93-2.17, n=3, I²<0) | aOR=1.21 (95% CI, 0.87-1.68, n=2, I²<0) | Rumrich 2016 |
| Obesity | OR= 1.64 (95% CI, 1.42–1.90, n=16, I²=UNK)  OR= 1.77 (95% CI 1.56- 2.03, n=15 I²=58%) | aOR= 1.52 (95% CI,1.36–1.70, n=16, I²=UNK)  aOR= 1.55 (95% CI 1.40 to 1.73, n=16, I²=24%) | Ino 2010  Rayfield  2017 |
| Overweight | OR=1.52 (95% CI, 1.36-1.69, n=14, I²= 49%)  OR= 1.43 (95% CI, 1.32-1.56, n=28, I²=64%) | aOR=1.50 (95% CI, 1.36-1.65, n=UNK, I²= 81%)  aOR= 1.37 (95% CI 1.28 to 1.46, n=31, I²=45%) | Oken 2008  Rayfield 2017 |
| Perinatal death | OR= 1.33 (95% CI, 1.23-1.45, n=35, I²=UNK) | aOR= 1.29 (95% CI, 1.20-1.39, n=12, I²=UNK) | Pineles 2016 |
| Renal tumors | Unadjusted or adjusted  OR= 0.95 (95% CI, 0.75-1.22, n=3, I²<0) | aOR= 0.94 (95% CI, 0.70-1.26, n=2, I²<0) | Rumrich 2016 |
| SIDS | Unadjusted or adjusted  OR= 2.25 (95% CI, 2.03–2.50, n=23, I²=76.6%) | Adjusted  aRR= 1.11 (95% CI, 1.01-1.22, n=10, I²=74.7%) | Zhang 2013 |
| Soft tissue tumors | OR= 0.88 (95% CI, 0.59-1.32, n=2, I²<0) | aOR= 0.88 (95% CI, 0.65-1.19, n=3, I²=0.0) | Rumrich 2016 |

# Additional file Table 10: Subgroup analyses: impact of study quality on results

| Condition | Low/Moderate Quality Studies | High Quality Studies | First Author & Year |
| --- | --- | --- | --- |
| Asthma | ***Aged 5-18;***  OR= 1.25 (95% CI, 1.00-1.5, n=4, I²=55.5%) | ***Aged 5-18;*** OR= 1.06 (95% CI, 0.67-1.68, n=4, I²=76.5%) | Burke 2012 |
| Atopic dermatitis | OR= 1.96 (95% CI, 1.50-2.64, n=UNK, I²=UNK) | OR= 0.88 (95% CI, 0.67-1.16, n=UNK, I²=UNK) | Kantor 2016 |
| Autism | Studies scored ≤6 indicated a positive association  OR= 1.65 (95% CI, 0.73-3.73, n=3, I²=83.1) | Studies >6 indicated null findings  OR= 1.02 (95% CI, 0.92-1.12, n=12, I²=60.4) | Rosen 2015  Tang 2015 |
| LBW | OR= 2.16 (95% CI, 1.68–2.77, n=14, I²=63.3%) | OR= 1.94 (95% CI, 1.68–2.24, n=16, I²=69.6%) | Pereira 2017 |
| Menarche age | ***All studies***  -0.092 year (95%CI, -0.160, -0.024, n=13, I²=UNK) | ***High quality studies only***  −0.116 year (95% CI,−0.214, −0.017, n=UNK) | Yermachenko 2015 |
| Middle ear disease | OR= 0.74 (0.24-2.27, n=2) | OR= 1.17 (95% CI, 0.95-1.44, n=4) | Jones 2012 |
| Miscarriage | OR= 1.23 (95% CI, 1.15-1.31, n=39) | OR= 1.28 (95% CI, 1.15-1.41, n=11) | Pineles 2014 |
| Overweight & obesity | ***All studies***  ***Overweight***  OR=1.07 (95% CI, 1.00-1.16, n=8, I²=0.00%) ***Obesity*** OR= 1.23 (95% CI, 1.10-1.38, n=6, I²=32.47%) | ***High quality studies only***  ***Overweight***  OR= 1.35 (95% CI, 1.15-1.58, n=4, I²=0.00%)  ***Obesity***  OR= 1.75 (95% CI, 1.10-2.80, n=2, I²=66.02%) | Reidel 2014 |
| Perinatal death | **Studies with highest risk of bias** OR= 1.34 (95% CI, 1.23-1.45, n=17, I²=UNK) | **Studies with lowest risk of bias** OR= 1.24 (95% CI, 1.18-1.30, n=2, I²=UNK) | Pineles 2016 |
| Placenta previa | OR= 1.4 (95% CI, 0.7-2.9, n=2, I²=UNK) | OR= 1.6 (95% CI, 1.5-1.8, n=5, I²=UNK) | Faiz 2003 |
| Preeclampsia | OR= 0.70 (95% CI, 0.67-0.72, n= 17)  RR= 0.66 (95% CI, 0.58–0.75, n=8, I²=64.9%) | OR= 0.67 (95% CI, 0.66-0.70, n=10)  RR= 0.67 (95% CI, 0.58–0.79, n=9, I²=95.0%) | Conde-Agudelo 1999  Wei 2015 |
| Stillbirth | OR= 1.49 (95% CI, 1.33-1.67, n=11, I²=UNK) | OR= 1.41 (95% CI, 1.28-1.55, n=14, I²=UNK) | Marufu 2015 |
| Wheezing | ***Aged 2 or younger;***  OR= 1.66 (95% CI, 0.93–2.96, n=5, I2 = 87.7%) ***Aged 3-4;*** OR= 1.35 (95% CI, 1.13-1.62,  n= 5, I²=72.6%) | ***Aged 2 or younger;***  OR= 1.35 (95% CI, 1.13–1.61, n=9, I²=85.3%) ***Aged 3-4;***  OR= 1.44 (95% CI, 0.92-2.25,  n= 3, I²=59.5%) | Burke 2012 |

# References

1. Ananth CV, Smulian JC, Vintzileos AM. Incidence of placental abruption in relation to cigarette smoking and hypertensive disorders during pregnancy: a meta-analysis of observational studies. *Obstet Gynecol.* 1999;93(4):622-628.

2. Ankum WM, Mol BWJ, Van der Veen F, Bossuyt PMM. Risk factors for ectopic pregnancy: A meta-analysis. *Fertility and Sterility.* 1996;65(6):1093-1099.

3. Antonopoulos CN, Sergentanis TN, Papadopoulou C, et al. Maternal smoking during pregnancy and childhood lymphoma: a meta-analysis. *International Journal of Cancer.* 2011;129(11):2694-2703.

4. Brion M-JA, Leary SD, Lawlor DA, Smith GD, Ness AR. Modifiable maternal exposures and offspring blood pressure: A review of epidemiological studies of maternal age, diet, and smoking. *Pediatric research.* 2008;63(6):593-598.

5. Burke H, Leonardi-Bee J, Hashim A, et al. Prenatal and Passive Smoke Exposure and Incidence of Asthma and Wheeze: Systematic Review and Meta-analysis. *Pediatrics.* 2012;129(4):735-744.

6. Castles A, Adams EK, Melvin CL, Kelsch C, Boulton ML. Effects of smoking during pregnancy. Five meta-analyses. *American journal of preventive medicine.* 1999;16(3):208-215.

7. Chao TK, Hu J, Pringsheim T. Prenatal risk factors for tourette syndrome: A systematic review. *BMC pregnancy and childbirth.* 2014;14 (1) (no pagination)(53).

8. Chrestani MA, Santos IS, Horta BL, Dumith SC, de Oliveira Dode MA. Associated factors for accelerated growth in childhood: a systematic review. *Maternal and child health journal.* 2013;17(3):512-519.

9. Chu P WHHSJYLJHWSJGYNX. Maternal smoking during pregnancy and risk of childhood neuroblastoma: Systematic review and meta-analysis. 2016.

10. Clifford A, Lang L, Chen R. Effects of maternal cigarette smoking during pregnancy on cognitive parameters of children and young adults: A literature review. *Neurotoxicology and Teratology.* 2012;34(6):560-570.

11. Conde-Agudelo A, Althabe F, Belizan JM, Kafury-Goeta AC. Cigarette smoking during pregnancy and risk of preeclampsia: a systematic review. *American Journal of Obstetrics & Gynecology.* 1999;181(4):1026-1035.

12. England L, Zhang J. Smoking and risk of preeclampsia: a systematic review. *Frontiers in bioscience : a journal and virtual library.* 2007;12:2471-2483.

13. Faiz AS, Ananth CV. Etiology and risk factors for placenta previa: An overview and meta-analysis of observational studies. *Journal of Maternal-Fetal and Neonatal Medicine.* 2003;13(3):175-190.

14. Fernandes M, Yang X, Li JY, Cheikh Ismail L. Smoking during pregnancy and vision difficulties in children: A systematic review. *Acta Ophthalmologica.* 2015;93(3):213-223.

15. Ferrante G, Antona R, Malizia V, Montalbano L, Corsello G, La Grutta S. Smoke exposure as a risk factor for asthma in childhood: a review of current evidence. *Allergy and asthma proceedings : the official journal of regional and state allergy societies.* 2014;35(6):454-461.

16. Flenady V, Koopmans L, Middleton P, et al. Major risk factors for stillbirth in high-income countries: A systematic review and meta-analysis. *The Lancet.* 2011;377(9774):1331-1340.

17. Hackshaw A, Charles R, Sadie B. Maternal smoking in pregnancy and birth defects: a systematic review based on 173 687 malformed cases and 11.7 million controls *Human Reproduction Update.* 2011;17(5):5.

18. Huang JS, Lee TA, Lu MC. Prenatal programming of childhood overweight and obesity. *Maternal & Child Health Journal.* 2007;11(5):461-473.

19. Huang Y, Huang J, Lan H, Zhao G, Huang C. A meta-analysis of parental smoking and the risk of childhood brain tumors. *PLoS ONE.* 2014;9 (7) (no pagination)(e102910).

20. Huang J, Zhu T, Qu Y, Mu D. Prenatal, perinatal and neonatal risk factors for intellectual disability: A systemic review and meta- Analysis. *PLoS ONE.* 2016;11 (4) (no pagination)(e0153655).

21. Huncharek MS, Kupelnick B, Klassen H. Maternal smoking during pregnancy and the risk of childhood brain tumors: A meta-analysis of 6566 subjects from twelve epidemiological studies. *Journal of Neuro-Oncology.* 2002;57(1):51-57.

22. Ino T. Maternal smoking during pregnancy and offspring obesity: Meta-analysis. *Pediatrics International.* 2010;52(1):94-99.

23. Jayes L, Haslam PL, Gratziou CG, et al. SmokeHaz: Systematic Reviews and Meta-analyses of the Effects of Smoking on Respiratory Health. *Chest.* 2016;150(1):164-179.

24. Jenabi E, Fereidooni B. The association between maternal smoking and hyperemesis gravidarum: a meta-analysis. *Journal of Maternal-Fetal and Neonatal Medicine.* 2017;30(6):693-697.

25. Jones LL, Hashim A, McKeever T, Cook DG, Britton J, Leonardi-Bee J. Parental and household smoking and the increased risk of bronchitis, bronchiolitis and other lower respiratory infections in infancy: systematic review and meta-analysis. *Respiratory Research.* 2011;12.

26. Jones LL, Hassanien A, Cook DG, Britton J, Leonardi-Bee J. Parental smoking and the risk of middle ear disease in children: a systematic review and meta-analysis. *Arch Pediatr Adolesc Med.* 2012;166(1):18-27.

27. Kantor R, Kim A, Thyssen JP, Silverberg JI. Association of atopic dermatitis with smoking: A systematic review and meta-analysis. *Journal of the American Academy of Dermatology.* 2016;75(6):1119-1125.e1111.

28. Lancaster CA, Gold KJ, Flynn HA, Yoo H, Marcus SM, Davis MM. Risk factors for depressive symptoms during pregnancy: a systematic review. *American Journal of Obstetrics and Gynecology.* 2010;202(1):5-14.

29. Latimer K, Wilson P, Kemp J, et al. Disruptive behaviour disorders: a systematic review of environmental antenatal and early years risk factors. *Child: care, health and development.* 2012;38(5):611-628.

30. Lee LJ, Lupo PJ. Maternal smoking during pregnancy and the risk of congenital heart defects in offspring: a systematic review and metaanalysis. *Pediatric cardiology.* 2013;34(2):398-407.

31. Linnet KM, Dalsgaard S, Obel C, et al. Maternal Lifestyle Factors in Pregnancy Risk of Attention Deficit Hyperactivity Disorder and Associated Behaviors: Review of the Current Evidence. *The American Journal of Psychiatry.* 2003;160(6):1028-1040.

32. Little J, Cardy A, Munger RG. Tobacco smoking and oral clefts: A meta-analysis. *Bulletin of the World Health Organization.* 2004;82(3):213-218.

33. Marufu TC, Ahankari A, Coleman T, Lewis S. Maternal smoking and the risk of still birth: systematic review and meta-analysis. *BMC public health.* 2015;15:239.

34. Mitchell EA, Milerad J. Smoking and the sudden infant death syndrome. *Reviews on Environmental Health.* 2006;21(2):81-103.

35. Nicoletti D, Appel LD, Siedersberger Neto P, Guimaraes GW, Zhang L. Maternal smoking during pregnancy and birth defects in children: a systematic review with meta-analysis. *Cadernos de saude publica.* 2014;30(12):2491-2529.

36. Oken E, Levitan E, Gillman M. Maternal smoking during pregnancy and child overweight: systematic review and meta-analysis. *International Journal of Obesity.* 2008;32:10.

37. Park SK, Kang D, McGlynn KA, et al. Intrauterine environments and breast cancer risk: meta-analysis and systematic review. *Breast cancer research : BCR.* 2008;10(1):R8.

38. Pereira PP, Da Mata FA, Figueiredo AC, de Andrade KR, Pereira MG. Maternal Active Smoking During Pregnancy and Low Birth Weight in the Americas: A Systematic Review and Meta-analysis. *Nicotine Tob Res.* 2017;19(5):497-505.

39. Pineles BL, Park E, Samet JM. Systematic Review and Meta-Analysis of Miscarriage and Maternal Exposure to Tobacco Smoke During Pregnancy. *American Journal of Epidemiology.* 2014;179(7):807-823.

40. Pineles BL, Hsu S, Park E, Samet JM. Systematic Review and Meta-Analyses of Perinatal Death and Maternal Exposure to Tobacco Smoke During Pregnancy. *American Journal of Epidemiology.* 2016;184(2):87-97.

41. Rayfield S, Plugge E. Systematic review and meta-analysis of the association between maternal smoking in pregnancy and childhood overweight and obesity. *Journal of epidemiology and community health.* 2017;71(2):162-173.

42. Riedel C, Schonberger K, Yang S, et al. Parental smoking and childhood obesity: higher effect estimates for maternal smoking in pregnancy compared with paternal smoking--a meta-analysis. *International Journal of Epidemiology.* 2014;43(5):1593-1606.

43. Rosen BN, Lee BK, Lee NL, Yang Y, Burstyn I. Maternal Smoking and Autism Spectrum Disorder: A Meta-analysis. *Journal of autism and developmental disorders.* 2015;45(6):1689-1698.

44. Rumrich IK, Viluksela M, Vahakangas K, Gissler M, Surcel HM, Hanninen O. Maternal Smoking and the Risk of Cancer in Early Life - A Meta-Analysis. *PLoS ONE [Electronic Resource].* 2016;11(11):e0165040.

45. Shah NR, Bracken MB. A systematic review and meta-analysis of prospective studies on the association between maternal cigarette smoking and preterm delivery. *American Journal of Obstetrics and Gynecology.* 2000;182(2):465-472.

46. Shobeiri F, Jenabi E. Smoking and placenta previa: a meta-analysis. *Journal of Maternal-Fetal and Neonatal Medicine.* 2017;30(24):2985-2990.

47. Shobeiri F, Masoumi SZ, Jenabi E. The association between maternal smoking and placenta abruption: a meta-analysis. *Journal of Maternal-Fetal and Neonatal Medicine.* 2017a;30(16):1963-1967.

48. Tang S, Wang Y, Gong X, Wang G. A Meta-Analysis of Maternal Smoking during Pregnancy and Autism Spectrum Disorder Risk in Offspring. *International Journal of Environmental Research & Public Health [Electronic Resource].* 2015;12(9):10418-10431.

49. Tsai MS, Chen MH, Lin CC, et al. Children's environmental health based on birth cohort studies of Asia. *The Science of the total environment.* 2017;609:396-409.

50. Tripathee S, Zhang J, Xiong M. Risk factors of microtia: A systematic review and meta-analysis. *European Journal of Plastic Surgery.* 2016;39(5):335-344.

51. Tuomisto J, Holl K, Rantakokko P, et al. Maternal smoking during pregnancy and testicular cancer in the sons: a nested case-control study and a meta-analysis. *European Journal of Cancer.* 2009;45(9):1640-1648.

52. Wang M, Wang ZP, Gong R, Zhao ZT. Maternal smoking during pregnancy and neural tube defects in offspring: a meta-analysis. *Childs Nervous System.* 2014;30(1):83-89.

53. Waylen AL, Metwally M, Jones GL, Wilkinson AJ, Ledger WL. Effects of cigarette smoking upon clinical outcomes of assisted reproduction: A meta-analysis. *Human Reproduction Update.* 2009;15(1):31-44.

54. Wei J, Liu CX, Gong TT, Wu QJ, Wu L. Cigarette smoking during pregnancy and preeclampsia risk: A systematic review and meta-analysis of prospective studies. *Oncotarget.* 2015;6(41):43667-43678.

55. Wendland EM, Pinto ME, Duncan BB, Belizán JM, Schmidt MI. Cigarette smoking and risk of gestational diabetes: a systematic review of observational studies. *BMC pregnancy and childbirth.* 2008;8:53.

56. Weng SF, Redsell SA, Swift JA, Yang M, Glazebrook CP. Systematic review and meta-analyses of risk factors for childhood overweight identifiable during infancy. *Archives of Disease in Childhood.* 2012;97(12):1019-1026.

57. Wyszynski DF, Duffy DL, Beaty TH. Maternal cigarette smoking and oral clefts: a meta-analysis. *Cleft Palate-Craniofacial Journal.* 1997;34(3):206-210.

58. Xuan Z, Zhongpeng Y, Yanjun G, et al. Maternal active smoking and risk of oral clefts: a meta-analysis. *Oral surgery, oral medicine, oral pathology and oral radiology.* 2016;122(6):680-690.

59. Yan K, Xu X, Liu X, et al. The associations between maternal factors during pregnancy and the risk of childhood acute lymphoblastic leukemia: A meta-analysis.[Erratum appears in Pediatr Blood Cancer. 2016 May;63(5):953-4; PMID: 26999072]. *Pediatric Blood & Cancer.* 2015;62(7):1162-1170.

60. Yermachenko A, Dvornyk V. A meta-analysis provides evidence that prenatal smoking exposure decreases age at menarche. *Reproductive Toxicology.* 2015;58:222-228.

61. Zhang D, Cui H, Zhang L, Huang Y, Zhu J, Li X. Is maternal smoking during pregnancy associated with an increased risk of congenital heart defects among offspring? A systematic review and meta-analysis of observational studies. *Journal of Maternal-Fetal & Neonatal Medicine.* 2017;30(6):645-657.

62. Zhang K, Wang X. Maternal smoking and increased risk of sudden infant death syndrome: a meta-analysis. *Legal medicine (Tokyo, Japan).* 2013;15(3):115-121.

63. Zwink N, Jenetzky E, Brenner H. Parental risk factors and anorectal malformations: Systematic review and meta-analysis. *Orphanet Journal of Rare Diseases.* 2011;6 (1) (no pagination)(25).
